# Diagnosing the Redox Character of Bond Activation by Main Group Centers: Reductive Addition vs Redox-Neutral Insertion as a Case Study

**DOI:** 10.1021/acs.inorgchem.6c01103

**Published:** 2026-04-24

**Authors:** Paola Belanzoni, Gerard Comas-Vilà, Diego Sorbelli

**Affiliations:** † Department of Chemistry, Biology and Biotechnology, University of Perugia, Via Elce di Sotto 8, 06123 Perugia, Italy; ‡ CNR Institute of Chemical Science and Technologies “Giulio Natta” (CNR-SCITEC), 06123 Perugia, Italy; § Pritzker School of Molecular Engineering, University of Chicago, 5640 S. Ellis Ave. Chicago, Illinois 60637, United States

## Abstract

The reductive addition of small molecules to low-valent main-group centers has recently started to emerge, yet their redox labeling is often inferred from qualitative electronegativity arguments and partial-charge trends. Here, we use a recent representative example, i.e., Zn–Zn bond cleavage by Si^II^ and Al^I^ main-group carbene analogues as a motivating case study and place it in a broader comparative computational analysis alongside conventionally oxidative H_2_ addition. We show that electronegativity- and partial-charge-based descriptors are strongly scheme- and scale-dependent and therefore insufficiently robust to diagnose redox character. In contrast, electron density- and wave-function-based metrics provide a consistent assignment across systems and reveal a central role of ligand electronics. Particularly, extensive ligand-assisted delocalization of the lone pair at Si leads to retention of a formal +2 oxidation state upon addition of Cp*ZnZnCp*, establishing the process as redox-neutral. More generally, our results identify ligand design as a practical handle to tune the reductive character of addition processes and provide a transferable, computation-led framework for assigning redox character in emerging main-group reactivity.

## Graphical abstract

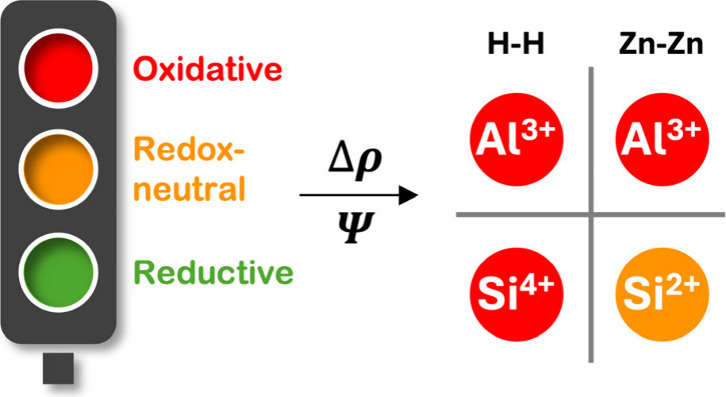

## Introduction

Oxidative addition and reductive elimination are two fundamental classes of reactions largely widespread across synthetic chemistry, as well as key mechanistic steps in many catalytic processes, including hydrogenations, hydrosilylation, hydroformylation, and cross-coupling reactions.^1,2^

The International Union of Pure and Applied Chemistry (IUPAC) defines oxidative addition as the insertion of a metal complex into a covalent bond (X-Y), increasing both the metal’s oxidation state and its coordination number by two.^3^ Addition processes that feature main-group compounds generally involve low-valent p-block elements (e.g., Si, Sn, Al, etc..) breaking strong bonds (e.g., H–H, C–H, B–B) and inserting themselves into the bond.^4–7^

While often exhibiting behavior similar to transition metals, these main-group compounds often challenge conventional views by resulting in reactions that cannot be formally described as strictly oxidative, sometimes being redox-neutral insertions (with no change in the oxidation state of the main-group element) or even “reductive” (decreasing oxidation state). The oxidative nature of H_2_ addition to several Ir complexes has been also questioned by Crabtree and co-workers.^8^ While the IUPAC does not provide a single, concise definition specifically for “reductive addition” as a standalone term in its Gold Book, it defines its components: “reductive” relates to gaining electrons or lowering oxidation state. Therefore, “reductive addition” describes a metal center gaining electrons (reduction) while incorporating atoms or groups (addition).

Recently, a striking unconventional case of reductive addition involving reactions of a Zn–Zn bond with main-group (E = Si, Al, Ga, In) carbene analogues has been reported.^9^ In particular, the addition of Cp*ZnZnCp* (Cp* = pentamethylcyclopentadienyl) to Si^II^(^Dipp^BDI-H) (Dipp = 2,6-*i*Pr_2_C_6_H_3_; ^Dipp^BDI-H = [H_2_C = C­(N-Dipp)-C­(H)=C­(Me)-NDipp]^2–^) has been proposed to “provide the most compelling case for a prototypical reductive addition process”.^9^ This reaction proceeds with complete breaking of the zinc–zinc bond and an increase in the coordination number of the central Si from two to four (Figure 1A).

**Figure 1:**
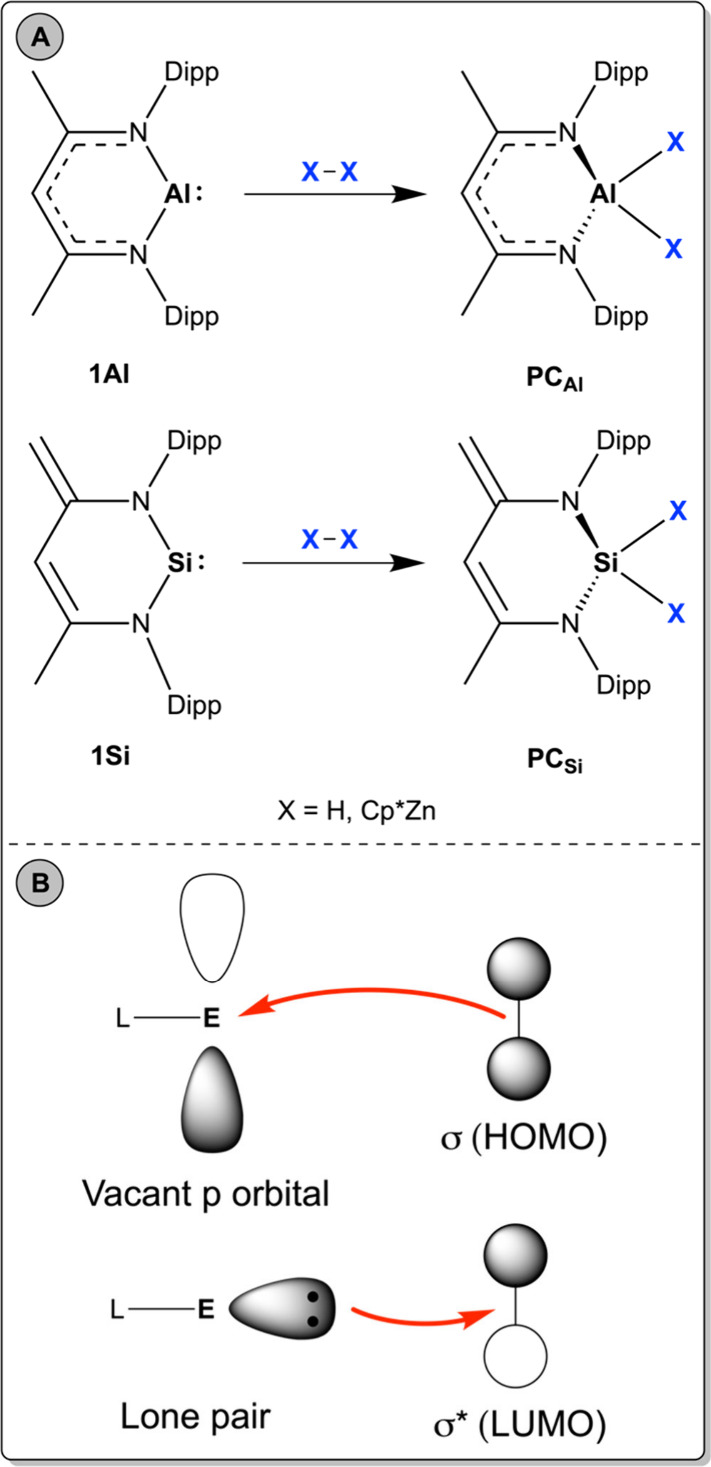
A) Oxidative addition of H_2_ and proposed reductive addition of Cp*ZnZnCp* to main-group carbene analogues Al^I^(NacNac) (**1Al**) and Si^II^(^Dipp^BDI-H) (**1Si**). B) Orbital interactions involved in the oxidative addition of H_2_ to main-group compounds (E = main-group element; L = ligand scaffold).

Conventional oxidative addition processes, such as the addition of H_2_ to a main-group element (E), involve electron density donation from the σ HOMO of H_2_ to an empty p orbital of E and a back-donation from an occupied orbital of E to the σ* LUMO of H_2_ (Figure 1B). The authors in ref 9 proposed an isolobal analogy between H–H and Zn–Zn bonds, with σ bonding and σ* antibonding molecular orbitals arising from the overlap of 4s–4s Zn atomic orbitals, similar to the 1s–1s overlap in H_2_. Therefore, the same mechanism is expected for the addition of Cp*ZnZnCp* to **1Al** and **1Si**.

In ref 9, the description of Cp*ZnZnCp* addition to the Si carbene is suggested as a reductive addition process mainly relying on electronegativity. For instance, the addition of H_2_ is defined as oxidative considering that the electronegativity of hydrogen is generally higher than that of most transition metals (M), resulting in the formation of polarized M^δ+^–H^δ−^ bonds. Following the same concept, silicon (χ_Si_ = 1.92) is more electronegative than zinc (χ_Zn_ = 1.59) on the Allen scale^10^ and as such the process should be defined as reductive from the perspective of silicon (formation of Si^δ−^–Zn^δ+^ bonds upon addition). In this view, the authors describe the corresponding addition process with the Al carbene analogue (Al^I^(NacNac), NacNac = [DippNC­(Me)­CHC­(Me)­NDipp]^−^) as oxidative or redox-neutral since the electronegativity of aluminum (χ_Al_ = 1.61) is very close to that of zinc. Atomic charges on Al and Si have also been computed to support the redox labeling, finding the charge on Si to decrease upon addition of Cp*ZnZnCp* (despite some numerical discrepancy), further corroborating the reductive character of the reaction. Following the philosophy of this work, other prototypical reductive addition processes have been reported,^11–13^ such as the addition of a Be–Be bond to a Sn^II^ center.^14^

It must be kept in mind, however, that neither electronegativities nor atomic charges are quantum mechanical observables. Both these quantities are method-dependent and have been shown to be unreliable in cases involving unconventional bonding motifs in main-group chemistry, due to the absence of a rigorous partitioning of electron density that would allow their unambiguous assignment.^15–18^

This issue motivates an unbiased assessment of whether these processes can truly be classified as reductive and whether electronegativities alone can serve as a general design principle in this framework. In this paper, we address these questions through a thorough computational investigation of the proposed unconventional reductive addition of Cp*ZnZnCp* to Si^II^(^Dipp^BDI-H) (**1Si**) and Al^I^(NacNac) (**1Al**) analogue in comparison with the corresponding classical oxidative addition of H_2_ (Figure 1A). We combine different and complementary electronic structure methods to describe both qualitatively and quantitatively the interaction between the substrates (H_2_ and Cp*ZnZnCp*) and **1Al** and **1Si** along the computed reaction paths, with the aim of elucidating the role of ligand scaffolds and main-group element nature on the redox character of the processes within an unbiased perspective.

## Results and Discussion

The Gibbs free energy profiles for the addition of Cp*ZnZnCp* and H_2_ to **1Al** and **1Si** were calculated using density functional theory (DFT, see the Computational Details section) and are compared in Figure 2. The optimized geometries of stationary points along the paths are reported in the Supporting Information (Figures S1–S5) and show good agreement with those calculated in ref 9 with a different computational setup.

**Figure 2:**
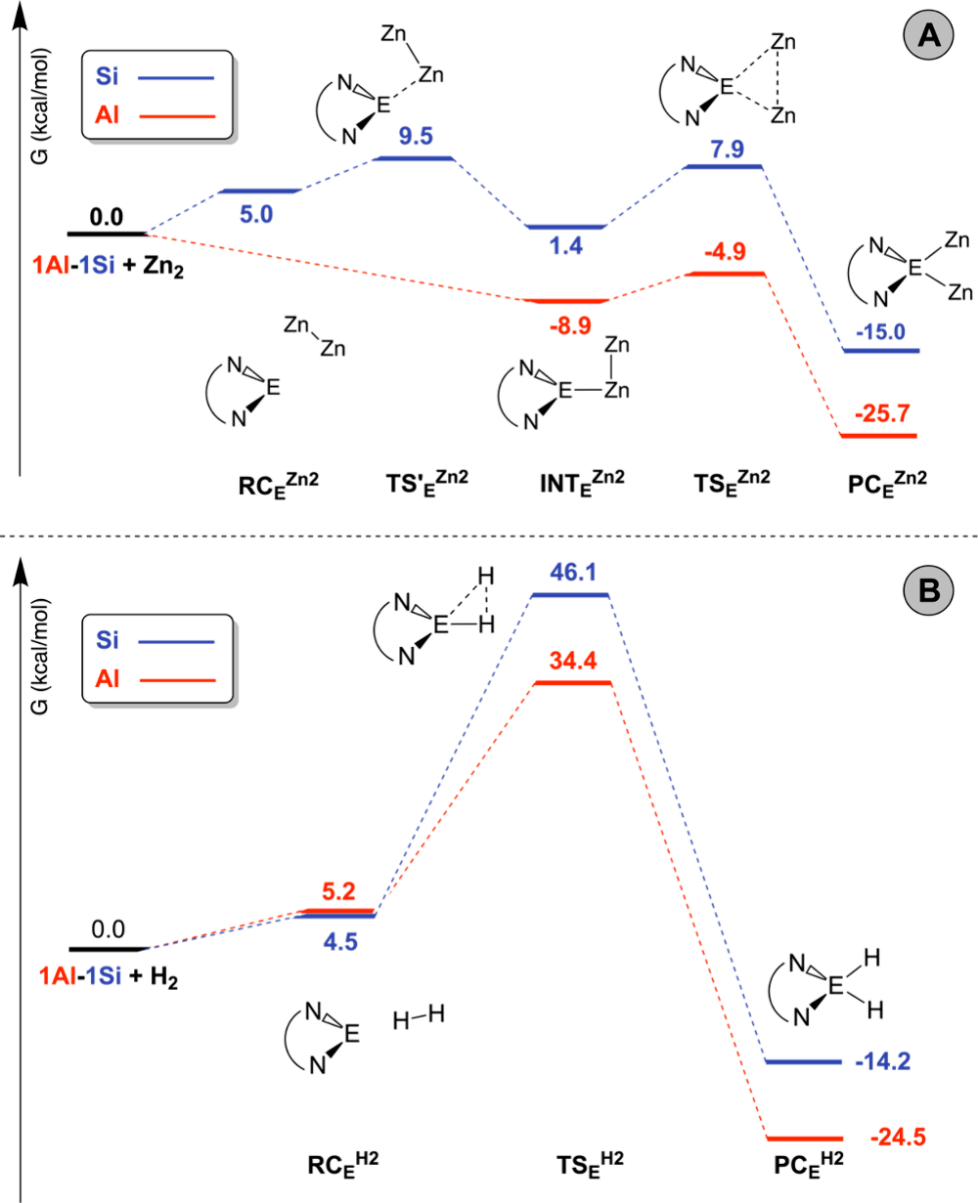
Reaction profiles for the addition of A) Cp*ZnZnCp* and B) H_2_ to **1Al** (red) and **1Si** (blue). Gibbs free energies (in kcal/mol) are relative to the separated reactants. E = Al, Si.

Reaction pathways for the addition of Cp*ZnZnCp* to **1Al** and **1Si** (Figure 2, panel A) are qualitatively similar to the corresponding pathways reported in ref 9 except for silicon, for which we find an earlier transition state (TS′_Si_^Zn2^) leading to the intermediate complex (INT_Si_^Zn2^) formation, in a slightly endergonic step, that was not previously detected. Similar to ref 9, for **1Al** the reaction occurs through formation of an intermediate INT_Al_^Zn2^ in a barrierless, exergonic step. From both INT_E_^Zn2^ intermediates, where one of the zinc centers is coordinated to Al or Si, the addition transition state TS_E_^Zn2^ is calculated at low energy (Δ*G* ^‡^ = 6.5 and 4.0 kcal/mol for Si and Al, respectively), featuring a three-centered structure with a substantial breaking of the Zn–Zn bond, and leading to the addition product (PC_E_^Zn2^) in a highly exergonic step. Differently, reaction pathways for the addition of H_2_ to **1Al** and **1Si** (Figure 2, panel B) show a one-step process, occurring through a concerted transition state TS_E_^H2^. The addition process, although highly exergonic, is expected to be kinetically unfeasible in mild conditions for both **1Al** and **1Si** (Δ*G* ^‡^ = 46.1 and 34.4 kcal/mol for Si and Al, respectively).

To elucidate the nature of the interaction of **1Al** and **1Si** with both substrates, we analyzed the electronic structure of the stationary points formed along the reaction paths. Particularly, we employ the Energy Decomposition Analysis (EDA)^19–21^ and Natural Orbitals for Chemical Valence (NOCV)^22,23^ (see Methodology in the Supporting Information) to analyze the **1Al**- and **1Si**-substrate interaction at all relevant transition states. This approach was previously shown by some of us to be particularly effective in interpreting the nature of unconventional reactive interactions without incurring partitioning issues (fragments are still well-defined at the selected transition states and no artifacts arising from bond breaking are introduced by their partitioning).^18,24–27^ For the reaction of H_2_ with **1Al** and **1Si**, the choice is straightforward (TS_E_^H2^), being a concerted mechanism. We chose, instead, TS_E_^Zn2^ for the addition of Cp*ZnZnCp* since they effectively represent the transition states wherein Zn–Zn bond breaking occurs.

At first glance, the EDA and NOCV approaches do not reveal remarkable differences among the four transition states. The most noticeable feature emerging from the EDA (Table S1 in the Supporting Information) is the more stabilizing interaction energy (Δ*E*_int_) associated with TS_E_^Zn2^ (−60.7 and −48.9 kcal/mol for Al and Si, respectively) compared to TS_E_^H2^ (−25.2 and −16.2 kcal/mol for Al and Si, respectively), which is fully consistent with the lower activation barriers found for the reactivity of **1Al** and **1Si** with Cp*ZnZnCp*. This difference between the two substrates is found to be mostly related to the stabilizing dispersive interactions at the TSs. The dispersion interaction energy (Δ*E*_disp_) is, as expected, practically negligible for TS_E_^H2^ (−2.2 and −1.3 kcal/mol for Al and Si, respectively), while the Cp* ligands are found to stabilize TS_E_^Zn2^ more efficiently (Δ*E*_disp_ is −23.6 and −27.4 kcal/mol for Al and Si, respectively), leading to a more negative overall interaction energy and, finally, to a lower activation barrier.

Similarly, the main NOCV pairs (Δρ_1_ and Δρ_2_, see Figure S6 in the Supporting Information) show similar dominant σ-type interactions, with comparable accumulation/depletion patterns on Al/Si and both substrates. A more in-depth analysis of Δρ_1_ and Δρ_2_, however, particularly in terms of decomposition of each deformation density, interestingly reveals distinct features related to the different nature of the main-group carbenes and of the two substrates. As discussed in the Methodology section and in the Supporting Information, the decomposition, in practice, allows one to obtain an objective picture of the molecular orbitals of each fragment involved in the reactivity.^22^

Using this approach, we decomposed Δρ_1_ and Δρ_2_ in terms of contribution of molecular orbitals of the two fragments to each deformation density. Concerning TS_E_^Zn2^ (see Figures S7–S14 in the Supporting Information for the complete decomposition panels), we find, as expected, that the HOMO of **1Al** (and the HOMO–1 of **1Si**), representing the lone pair on Al (Si), is involved in the charge transfer to the σ* LUMO of H_2_ and Cp*ZnZnCp* in both Δρ_1_ and Δρ_2_ (Figure 3, panel A). Analogously, a back-transfer is observed from the bonding HOMO of H_2_ (HOMO–4 of Cp*ZnZnCp*) to the LUMO+1 of **1Al** and LUMO of **1Si**, representing the vacant carbene-like p orbital perpendicular to the ring plane (Figure 3B). These findings confirm the orbital analogy between **1Al** and **1Si** (and H_2_ and Cp*ZnZnCp*) discussed earlier.

**Figure 3:**
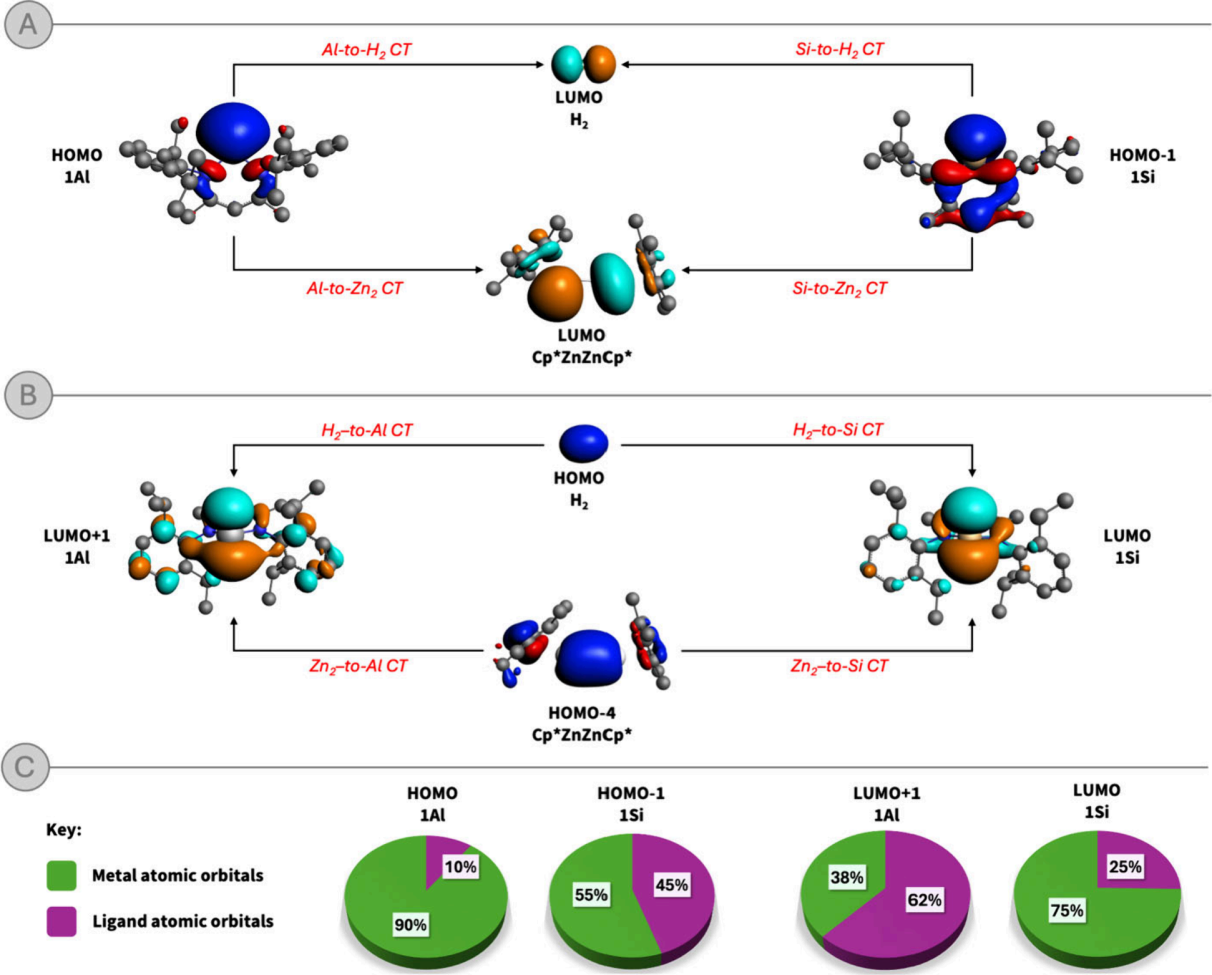
A,B) Frontier molecular orbitals involved in the reactivity between **1Al** and **1Si** with H_2_ and Cp*ZnZnCp*. The isovalue is 30 me/a_0_^3^ for all isosurfaces. C) Atomic orbital character (in percentage) of the frontier molecular orbitals of **1Al** and **1Si**.

Further inspection of the isosurfaces in Figure 3, however, highlights several interesting features that distinguish the species involved in this reactivity, providing a rationale to differentiate between the nature of the reactive processes. First, we find that the isolobal analogy between H_2_ and Zn_2_ might not fully hold. The σ and σ* MOs of Cp*ZnZnCp*, while qualitatively analogous in nature to those of H_2_, are partially delocalized on the Cp* rings, indicating possible ligand involvement in the reactivity. Consistently, the accumulation/delocalization patterns on the dizinc fragment in both NOCV deformation densities highlight (albeit small) contributions from both Cp* ligands that may influence the electrons transferred between the two fragments, an effect for which, clearly, no analogy with dihydrogen exists.

Additionally, despite similar orbital character, it is immediately evident from Figure 3 that the frontier molecular orbitals of **1Al** and **1Si** are quantitatively different. The HOMO (lone pair) of **1Al** is almost fully localized on the Al center, with poor delocalization over the ligand scaffold. From a perspective of MO character in terms of atomic orbitals, the composition of the HOMO of **1Al** arises almost completely from Al atomic orbitals (90%, Figure 3C left), with the ligand’s atomic orbitals contributing for only 10%. Instead, the lone pair on **1Si** is largely delocalized on the ligand (the ligand contribution to the HOMO–1 of **1Si** amounts to 55%, Figure 3C center-left). Inversely, the LUMO+1 of **1Al** is more delocalized over the ligand backbone (62% ligand character, Figure 3C, right-center) compared to the LUMO of **1Si** (75% Si character, Figure 3C, right).

The different ligand delocalization schemes in **1Al** and **1Si** likely arise from the different conjugation patterns of the ligand backbone (see Figure 1A). In **1Si**, the dianionic and strongly donating [^Dipp^BDI-H]^2–^ ligand also bears an additional terminal methylene unit which favors delocalization. As a result of this electronic structure, the occupied orbitals of the 2-fold deprotonated β-diketiminate-type scaffold mix more effectively with the Si-centered lone pair, yielding an overall delocalized donor MO at **1Si**. Notably, this effect was also previously shown and discussed by some of us for carbenes (C­(^Dipp^BDI-H) is isoelectronic to **1Si**), where its extensive backbone delocalization affects the lone pair strength and basicity.^27,28^ In this context, while the reactive MOs of **1Al** fully fill the expected brief for a standard oxidative process (Figure 1A), the highly delocalized lone pair in **1Si** suggests (i) that the lone pair is less available for reactivity and (ii) that there is a lower tendency of the Si center to undergo oxidation, with the ligand being actively involved in the reactive process. In other words, our results suggest that the addition of Cp*ZnZnCp* to **1Si** might not fully be considered oxidative because of the relevant cooperative effect of the ligand backbone.

We turn to employ more quantitative measures with the aim of assessing whether this process can be really considered reductive. A comparative analysis of the atomic electronegativities represents the formal approach to assess the reductive/oxidative character of an addition process. Following the Allen electronegativity scale, for instance, the electronegativity of H (2.30) is larger than that of Al (1.61) and Si (1.92), suggesting that the addition of H_2_ to either system should be oxidative (Figure 4A). The electronegativity of Zn (1.59), instead, is comparable to that of Al and smaller than that of Si, indicating that, formally, the addition of **1Al** and **1Si** into the Zn–Zn bond cannot be considered oxidative. Two issues, however, might arise with electronegativities. First, it is known that atomic electronegativities can be misleading when “unconventional” covalent bonds are formed,^18,27^ because molecular moieties (or ligand scaffolds) can perturb the electronic structure of the atomic center of interest, effectively yielding a “molecular” electronegativity that vastly differs from the atomic one. Second, electronegativities are highly scale-dependent: as shown in Figure 4A (and Figure S15 in the Supporting Information), a comparative analysis of the electronegativities using other scales does not necessarily yield the same outcome. For instance, in the Allred–Rochow scale,^29^ the electronegativity of Zn (1.66) is only marginally smaller than that of Si (1.74) and higher than that of Al (1.47), while in the Sanderson scale,^30^ the electronegativity of Zn (2.33) is higher than that of Si (2.14). This indicates that labeling the insertion into Zn–Zn bonds of **1Al** and **1Si** as oxidative, redox-neutral or reductive is equally justifiable across different scales.

**Figure 4:**
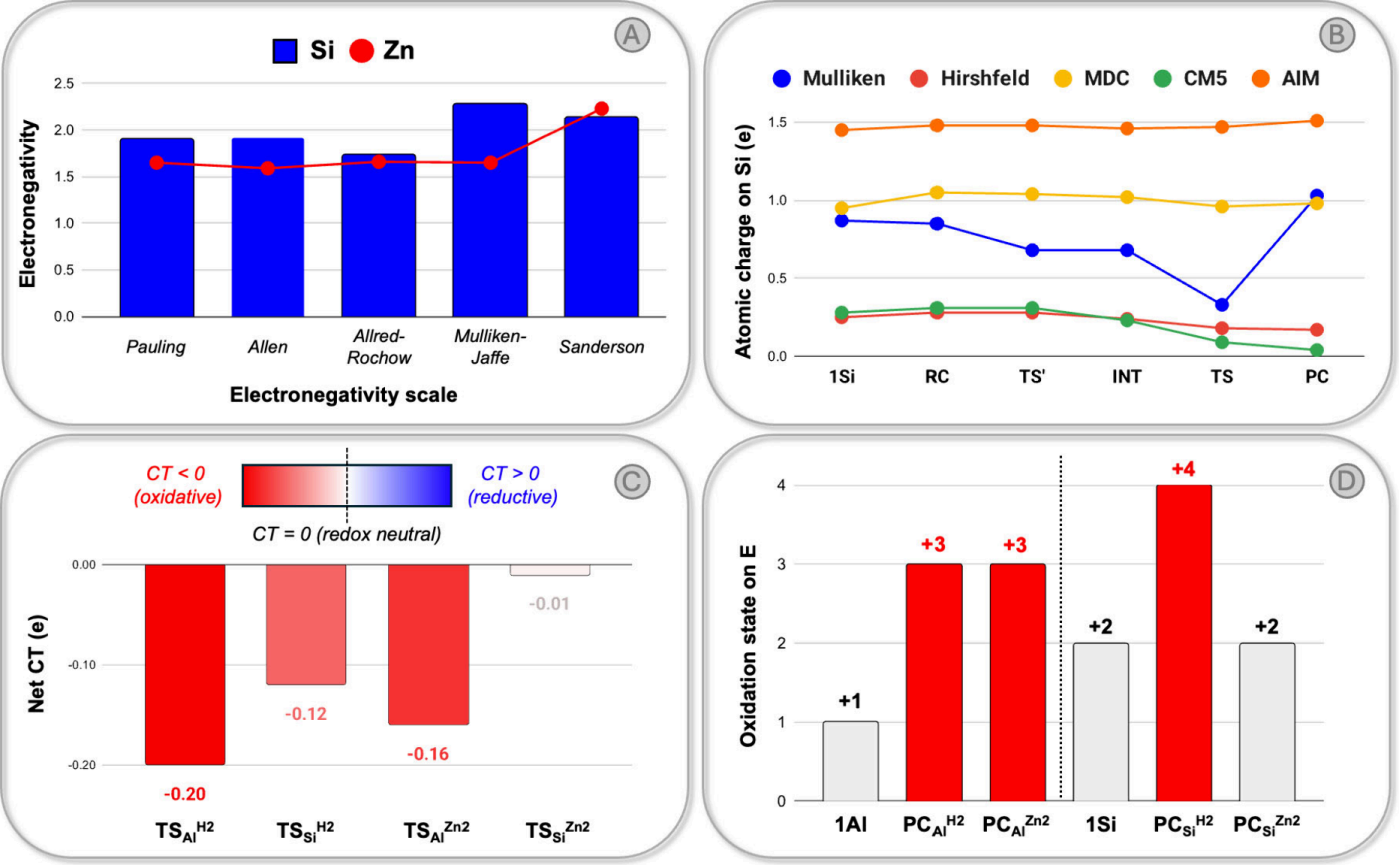
A) Comparison of the electronegativities of Si (blue bars) and Zn (red dots) across different scales. B) Computed atomic charges on Si along the path for the reaction of **1Si** with Cp*ZnZnCp* using different methods. C) Charge transfer (CT, in electrons) between **1Al**/**1Si** and H_2_/Cp*ZnZnCp* at the relative transition states TS_E_^X2^. D) Effective oxidation states of Al and Si in the main-group carbenes (**1Al**, **1Si**) and in the insertion products PC_E_^X2^.

A similar ambiguity emerges from atomic charge analyses (Figure 4B and Figure S16 in the Supporting Information). Atomic charges are not well and uniquely defined, so their absolute and relative values are highly dependent on the computational accuracy (e.g., basis set) and the method-dependent partitioning scheme used. We computed the atomic charges on Al and Si for all stationary points along both reaction paths, using five different approaches and a large QZ4P basis set. As expected, different methods yield different results: while, trendwise, the addition of H_2_ leads to an almost consistent increase of atomic charge on both Al and Si, the charges on both Zn–Zn insertion products are highly method-dependent and can support the interpretation of oxidative, redox-neutral, or reductive character depending on the method. Therefore, electronegativity and partial charges cannot unambiguously diagnose the redox character of the processes.

Motivated by the inherent biases of the descriptors discussed so far, we employed electron density- and wave-function-based approaches that could provide an unbiased overview of the redox character of the processes. At first, we used the Charge Displacement (CD) approach^31–33^ (see the Methodology section in the Supporting Information for details), an electron-density-based approach used to quantify electron flow between well-defined fragments at the transition states, providing an exact measure of the magnitude and direction of the charge transfer.^24–26^ In all cases, fragments are defined so that a positive charge transfer (CT) value indicates net electron flow from H_2_/Cp*ZnZnCp* toward **1Al**/**1Si**, thereby describing a reductive process, while a negative CT indicates the opposite.

As shown in Figure 4C, no unambiguous reference to reductive character is found from the CD analysis. For the addition of H_2_, the computed CT values are negative for both TSs (−0.20 e and −0.12 e for Al and Si, respectively), indicating that the process can be interpreted as a conventional oxidative addition, with electrons being transferred to H_2_. Interestingly, for the insertion of **1Al** into the Zn–Zn bond, the application of the CD approach at TS_Al_^Zn2^ reveals a negative CT value of −0.16 e, which indicates that the addition of Cp*ZnZnCp* on Al can be formally considered as oxidative as well. Finally, we find an almost neutral CT value (−0.01 e) for TS_Si_^Zn2^, indicating essentially no net flow of electrons between **1Si** and Cp*ZnZnCp* and strongly suggesting a redox-neutral character for the process.

To quantify redox changes more directly, we employed the Effective Oxidation State (EOS) analysis,^34^ a wave-function-based approach that allows oxidation states to be assigned in a fragment-resolved manner beyond formal electron-counting schemes (see the Supporting Information for details).

To monitor oxidation-state variations along the reaction pathway, we first applied EOS analysis to the reference **1Al** and **1Si** compounds. The EOS analysis partitions the occupied effective fragment orbitals between the main-group center and the ligand scaffold, allowing electrons to be consistently assigned to each fragment and thus enabling a direct assessment of the oxidation state of Al and Si in the reference compounds (see Table S2 in the Supporting Information and discussion therein for details). As a result, the computed oxidation states (OSs) of **1Al** and **1Si** (Figure 4D and Tables S2 and S3 and discussion therein) are consistent with the expected OSs, namely +1 for the Al center in **1Al** and +2 for the Si center in **1Si**. The EOS analysis of PC_E_^H2^ reveals that, upon addition, both Al and Si centers undergo full oxidation, assigning +3 and +4 oxidation states to Al and Si, respectively, while each hydrogen ligand is assigned an anionic (−1) character, consistent with full oxidation. We note that describing the coordinated Cp*Zn unit as an anionic fragment, [Cp*Zn]^−^, formally corresponds to a zerovalent zinc center, since Cp* itself is a monoanionic ligand. Interestingly, an analogous picture emerges for PC_Al_^Zn2^, where Al exhibits a +3 oxidation state and a −1 OS is assigned to each ZnCp* moiety, in close analogy with dihydrogen addition and clearly indicating an oxidative process. In contrast, PC_Si_^Zn2^ displays a distinct behavior: the EOS analysis assigns a +2 oxidation state to Si (and OS of 0 for each Cp*Zn moiety), suggesting that the addition proceeds via a redox-neutral process distinct from the behavior observed for the other compounds studied here, and corresponds to an intermediate scenario between full oxidation and full reduction, entirely consistent with the analyses presented above.

## Conclusions

In summary, the analyses presented in this paper indicate that describing Cp*ZnZnCp* addition to **1Si** (and **1Al**) as “reductive” is not justified when the redox character is assessed through quantitative and unbiased electronic-structure descriptors. The key differentiating feature is the ligand-enabled electronic structure of **1Si**: pronounced delocalization of the nominal Si lone pair into the [^Dipp^BDI-H]^2–^ scaffold reduces its availability for oxidation and provides a natural, mechanistic explanation for the observed behavior. Accordingly, multiple independent density-based and wave-function-based metrics consistently identify the reaction of Cp*ZnZnCp* with **1Si** reaction as a redox-neutral insertion, with Si retaining a +2 oxidation state in the product, while electronegativity arguments and partial charges remain nondiagnostic due to their scale, basis, and partitioning dependence.

More generally, the comparative assessment of Cp*ZnZnCp* addition and H_2_ activation reactions illustrates that establishing redox character in main-group bond activation requires (i) descriptors that are robust to methodological choices and (ii) explicit consideration of the ligand electronic structure and its influence on the reactivity. When analyzed through this lens, “reductive addition” and “redox-neutral insertion” are best viewed as limiting cases connected by a tunable continuum that can be shifted through ligand electronics. This work provides a practical framework for interpreting emerging reactivity reports and for designing new systems with tailored redox behavior: by modulating the extent of ligand-assisted delocalization, one can rationally steer bond activation toward more oxidative or more reductive regimes. We expect this approach to be broadly applicable to other low-valent main-group reagents and bond activation motifs, facilitating consistent mechanistic classification and predictive reactivity control.

## Computational Details

All geometry optimizations and frequency calculations on optimized structures (minima with zero imaginary frequencies and transition states with one imaginary frequency) for both the Cp*ZnZnCp* and H_2_ additions into the [Si^II^(^Dipp^BDI-H)] and [Al^I^(NacNac)] complexes reactions have been carried out using the Amsterdam Density Functional (ADF) code^35^ in combination with the related Quantum-regions Interconnected by Local Description (QUILD) program.^36^ The PBE^37^ GGA exchange–correlation (XC) functional, the TZ2P basis set with a small frozen core approximation for all atoms, the ZORA Hamiltonian^38–40^ for treating scalar relativistic effects and the Grimme’s D3-BJ dispersion correction were used.^41,42^ Solvent effects were modeled employing the Conductor-like Screening Model (COSMO) with the default parameters for benzene as implemented in the QUILD code.^43^ The same computational setup has also been used for the EDA and CD-NOCV calculations. This computational protocol has been used by some of us to study the [^t^Bu_3_PAuAl­(NON)] and [^t^Bu_3_PAuCO_2_Al­(NON)] complexes^27^ as well as in ref 28 to investigate the differences in coordination chemistry between aluminyl and carbene ligands.

The atomic charges were calculated using different schemes as implemented in the ADF code, that is Mulliken charges,^44^ Hirshfeld charges,^45^ Multipole Derived Charge (MDC) analysis,^46^ Charge Model Five (CM5) analysis,^47^ and Bader’s Atoms in Molecules (AIM) analysis.^48,49^ All charges have been calculated at the PBE-D3 level using a large QZ4P basis set. The atomic electronegativities from the Mulliken,^50^ Allen,^10^ Allred–Rochow,^29^ Pauling,^51^ and Sanderson^30^ scales for Al, H, Zn and Si have been reported as well.

Spin-resolved effective fragment orbitals and subsequent effective oxidation state analysis were performed at the PBE/def2TZVP level of theory with the APOST-3D program,^52^ using the topological fuzzy Voronoi cells (TFVC) real-space partitioning for the atomic definitions.^53^

## Supplementary Material



## Notes

Methodology, structures (images and xyz formats) of the stationary points along the reaction paths with related additional discussion, tables and figures with additional EDA and NOCV results, analysis of the atomic electronegativities, atomic charges, EOS data with related discussion, and atomic coordinates (PDF)

## References

[1] Stahl S. S. (2010). Organotransition
Metal Chemistry: From Bonding to Catalysis. J. Am. Chem. Soc..

[2] Firsan S. J., Sivakumar V., Colacot T. J. (2022). Emerging Trends in Cross-Coupling:
Twelve-Electron-Based L1Pd(0) Catalysts, Their Mechanism of Action,
and Selected Applications. Chem. Rev..

[3] Muller P. (1994). Glossary of
Terms Used in Physical Organic Chemistry (IUPAC Recommendations 1994). Pure Appl. Chem..

[4] Chu T., Nikonov G. I. (2018). Oxidative Addition
and Reductive Elimination at Main-Group
Element Centers. Chem. Rev..

[5] Asay M., Jones C., Driess M. (2011). N-Heterocyclic
Carbene Analogues
with Low-Valent Group 13 and Group 14 Elements: Syntheses, Structures,
and Reactivities of a New Generation of Multitalented Ligands. Chem. Rev..

[6] Liu Y., Li J., Ma X., Yang Z., Roesky H. W. (2018). The Chemistry of
Aluminum­(I) with *β*-Diketiminate Ligands and
Pentamethylcyclopentadienyl-Substituents: Synthesis, Reactivity and
Applications. Coord. Chem. Rev..

[7] He M., Hu C., Wei R., Wang X.-F., Liu L. L. (2024). Recent Advances
in the Chemistry of Isolable Carbene Analogues with Group 13–15
Elements. Chem. Soc. Rev..

[8] Crabtree R. H., Quirk J. M. (1980). Oxidative versus
Reductive Additions: A ^13^C NMR Study of H_2_,
HX (X = Cl, Br, OR I), and Cl_2_ Additions to Some Iridium­(I)
Complexes. J.
Organomet. Chem..

[9] Yang W., White A. J. P., Crimmin M. R. (2025). Reactions of a Zn–Zn
Bond
with Main Group Carbene Analogues as a Prototypical Case of Reductive
Addition. Nat. Synth..

[10] Allen L. C. (1989). Electronegativity
Is the Average One-Electron Energy of the Valence-Shell Electrons
in Ground-State Free Atoms. J. Am. Chem. Soc..

[11] Ohmura T., Morimasa Y., Suginome M. (2015). Organocatalytic Diboration Involving
“Reductive Addition” of a Boron–Boron σ-Bond
to 4,4′-Bipyridine. J. Am. Chem. Soc..

[12] Neeve E. C., Geier S. J., Mkhalid I. A. I., Westcott S. A., Marder T. B. (2016). Diboron­(4)
Compounds: From Structural Curiosity to Synthetic Workhorse. Chem. Rev..

[13] Griffin L. P., Boronski J. T. (2025). Alkaline Earth Metals:
Heterometallic Bonding. Dalton Trans..

[14] Dietz M., Boronski J. T., Swarbrook A. M., Aldridge S. (2025). Breaking Bonds at Tin­(II):
Reductive or Oxidative Addition?. Angew. Chem.,
Int. Ed..

[15] Sproul G. D. (2020). Evaluation
of Electronegativity Scales. ACS Omega.

[16] Leach I. F., Sorbelli D., Belpassi L., Belanzoni P., Havenith R. W. A., Klein J. E. M. N. (2022). How Reduced Are Nucleophilic Gold
Complexes?. Dalton Trans..

[17] Riemann A., Rankin L., Henry D. (2024). Atomic Charge
Dependency of Spiropyran/Merocyanine
Adsorption as a Precursor to Surface Isomerization Reactions. ACS Omega.

[18] Sorbelli D., Belpassi L., Belanzoni P. (2024). Cooperative
Small Molecule Activation
by Apolar and Weakly Polar Bonds through the Lens of a Suitable Computational
Protocol. Chem. Commun..

[19] Morokuma K. (1971). Molecular
Orbital Studies of Hydrogen Bonds. III. C = O···H–O
Hydrogen Bond in H_2_CO···H_2_O and
H_2_CO···2H_2_O. J. Chem. Phys..

[20] Ziegler T., Rauk A. (1977). On the Calculation
of Bonding Energies by the Hartree Fock Slater
Method. Theor. Chim. Acta.

[21] Zhao L., von Hopffgarten M., Andrada D. M., Frenking G. (2018). Energy Decomposition
Analysis. WIREs Comput. Mol. Sci..

[22] Mitoraj M., Michalak A. (2007). Natural Orbitals for
Chemical Valence as Descriptors
of Chemical Bonding in Transition Metal Complexes. J. Mol. Model..

[23] Michalak A., Mitoraj M., Ziegler T. (2008). Bond Orbitals
from Chemical Valence
Theory. J. Phys. Chem. A.

[24] Sorbelli D., Belanzoni P., Belpassi L., Lee J.-W., Ciancaleoni G. (2022). An ETS-NOCV-Based
Computational Strategies for the Characterization of Concerted Transition
States Involving CO_2_. J. Comput.
Chem..

[25] Sorbelli D., Belpassi L., Belanzoni P. (2023). Widening the Landscape of Small Molecule
Activation with Gold-Aluminyl Complexes: A Systematic Study of E–H
(E = O, N) Bonds, SO_2_ and N_2_O Activation. Chem. – Eur. J..

[26] Sorbelli D., Belpassi L., Belanzoni P. (2022). Mechanistic
Study of Alkyne Insertion
into Cu–Al and Au–Al Bonds: A Paradigm Shift for Coinage
Metal Chemistry. Inorg. Chem..

[27] Sorbelli D., Belpassi L., Belanzoni P. (2021). Reactivity of a Gold-Aluminyl Complex
with Carbon Dioxide: A Nucleophilic Gold?. J.
Am. Chem. Soc..

[28] Sorbelli D., Belpassi L., Belanzoni P. (2022). Unraveling
Differences in Aluminyl
and Carbene Coordination Chemistry: Bonding in Gold Complexes and
Reactivity with Carbon Dioxide. Chem. Sci..

[29] Allred A.
L., Rochow E. G. (1958). A Scale
of Electronegativity Based on Electrostatic
Force. J. Inorg. Nucl. Chem..

[30] Sanderson R. T. (1951). An Interpretation
of Bond Lengths and a Classification of Bonds. Science.

[31] Belpassi L., Infante I., Tarantelli F., Visscher L. (2008). The Chemical Bond between
Au­(I) and the Noble Gases. Comparative Study of NgAuF and NgAu^+^ (Ng = Ar, Kr, Xe) by Density Functional and Coupled Cluster
Methods. J. Am. Chem. Soc..

[32] Bistoni G., Rampino S., Tarantelli F., Belpassi L. (2015). Charge-Displacement
Analysis via Natural Orbitals for Chemical Valence: Charge Transfer
Effects in Coordination Chemistry. J. Chem.
Phys..

[33] Bistoni G., Belpassi L., Tarantelli F. (2016). Advances in Charge Displacement Analysis. J. Chem. Theory Comput..

[34] Ramos-Cordoba E., Postils V., Salvador P. (2015). Oxidation
States from Wave Function
Analysis. J. Chem. Theory Comput..

[35] te
Velde G., Bickelhaupt F. M., Baerends E. J., Fonseca
Guerra C., van Gisbergen S. J. A., Snijders J. G., Ziegler T. (2001). Chemistry
with ADF. J. Comput. Chem..

[36] Swart M., Bickelhaupt F. M. (2008). QUILD:
QUantum-Regions Interconnected by Local Descriptions. J. Comput. Chem..

[37] Perdew J. P., Burke K., Ernzerhof M. (1996). Generalized
Gradient Approximation
Made Simple. Phys. Rev. Lett..

[38] Lenthe E. van, Baerends E. J., Snijders J. G. (1993). Relativistic Regular Two-component
Hamiltonians. J. Chem. Phys..

[39] van
Lenthe E., Baerends E. J., Snijders J. G. (1994). Relativistic Total
Energy Using Regular Approximations. J. Chem.
Phys..

[40] van
Lenthe E., Ehlers A., Baerends E.-J. (1999). Geometry Optimizations
in the Zero Order Regular Approximation for Relativistic Effects. J. Chem. Phys..

[41] Grimme S., Antony J., Ehrlich S., Krieg H. (2010). A Consistent and Accurate
Ab Initio Parametrization of Density Functional Dispersion Correction
(DFT-D) for the 94 Elements H-Pu. J. Chem. Phys..

[42] Grimme S., Ehrlich S., Goerigk L. (2011). Effect of
the Damping Function in
Dispersion Corrected Density Functional Theory. J. Comput. Chem..

[43] Pye C. C., Ziegler T. (1999). An Implementation of
the Conductor-like Screening Model
of Solvation within the Amsterdam Density Functional Package. Theor. Chem. Acc..

[44] Mulliken R. S. (1955). Electronic
Population Analysis on LCAO–MO Molecular Wave Functions. I. J. Chem. Phys..

[45] Hirshfeld F. L. (1977). Bonded-Atom
Fragments for Describing Molecular Charge Densities. Theor. Chim. Acta.

[46] Swart M., van Duijnen P. Th, Snijders J. G. (2001). A Charge Analysis Derived from an
Atomic Multipole Expansion. J. Comput. Chem..

[47] Marenich A. V., Jerome S. V., Cramer C. J., Truhlar D. G. (2012). Charge Model 5:
An Extension of Hirshfeld Population Analysis for the Accurate Description
of Molecular Interactions in Gaseous and Condensed Phases. J. Chem. Theory Comput..

[48] Rodríguez J. I., Köster A. M., Ayers P. W., Santos-Valle A., Vela A., Merino G. (2009). An Efficient
Grid-Based Scheme to
Compute QTAIM Atomic Properties without Explicit Calculation of Zero-Flux
Surfaces. J. Comput. Chem..

[49] Rodríguez J. I., Bader R. F. W., Ayers P. W., Michel C., Götz A. W., Bo C. (2009). A High Performance
Grid-Based Algorithm for Computing QTAIM Properties. Chem. Phys. Lett..

[50] Mulliken R. S. (1934). A New Electroaffinity
Scale; Together with Data on Valence States and on Valence Ionization
Potentials and Electron Affinities. J. Chem.
Phys..

[51] Pauling L. (1932). The Nature
of the Chemical Bond. IV. The Energy of Single Bonds and the Relative
Electronegativity of Atoms. J. Am. Chem. Soc..

[52] Salvador P., Ramos-Cordoba E., Montilla M., Pujal L., Gimferrer M. (2024). APOST-3D:
Chemical Concepts from Wavefunction Analysis. J. Chem. Phys..

[53] Salvador P., Ramos-Cordoba E. (2013). Communication: An Approximation to Bader’s Topological
Atom. J. Chem. Phys..

