# Supplementary material for: Diagnosing the Redox Character of Bond Activation by Main Group Centers: Reductive Addition vs Redox-Neutral Insertion as a Case Study
Source: Inorg Chem. 2026 Apr 24;65(18):9711–8. doi: 10.1021/acs.inorgchem.6c01103 (PMC13169383; doi:10.1021/acs.inorgchem.6c01103)
Supplement: Supplementary file 1 [file ic6c01103_si_001.pdf]

Supporting Information

Diagnosing the redox character of bond activation by main group centers: reductive addition vs. redox-neutral insertion as a case study

Paola Belanzoni,[†,§,*] Gerard Comas-Vilà[†] and Diego Sorbelli[†,¢,*]

†Department of Chemistry, Biology and Biotechnology, University of Perugia, Via Elce di Sotto 8, 06123, Perugia, Italy. Paola Belanzoni email: paola.belanzoni@unipg.it; Diego Sorbelli email: diego.sorbelli@unipg.it.

§CNR Institute of Chemical Science and Technologies "Giulio Natta" (CNR-SCITEC), 06123 Perugia, Italy.

¢Pritzker School of Molecular Engineering, University of Chicago, 5640 S. Ellis Ave. Chicago, IL, 60637, United States

Table of Contents

**Methodology**	**S2**
**Figures S1-S5.** Stationary points and additional discussion	**S8**
**Table S1.** Energy Decomposition Analysis	**S13**
**Figures S6-S14.** NOCV deformation densities and decomposition panels	**S14**
**Figures S15-16.** Comparative electronegativity and atomic charge analyses	**S23**
**Tables S2-S3.** EOS analysis and additional discussion	**S25**
**References**	**S27**
**xyz geometries**	**S30**

Methodology

- **Energy Decomposition Analysis (EDA) and Extended Transition State (ETS) - NOCV approach**

In this work the Energy Decomposition Analysis (EDA)[1–3] has been applied to get preliminary insights into the interaction between [Cp*ZnZnCp*] and the [Si^{II}(^{Dipp}BDI-H)] and [Al^{I}(NacNac)] complexes and between [H_2] and the [Si^{II}(^{Dipp}BDI-H)] and [Al^{I}(NacNac)] complexes in the transition states TS_E^{Zn2} and TS_E^{H2}, respectively. With this approach, the interaction energy between the [Si^{II}(^{Dipp}BDI-H)] and [Cp*ZnZnCp*], [Al^{I}(NacNac)] and [Cp*ZnZnCp*], [Si^{II}(^{Dipp}BDI-H)] and [H_2] and [Al^{I}(NacNac)] and [H_2] fragments can be decomposed in different contributions as follows:

$$\Delta E_{\mathrm{int}} = \Delta E_{Pauli} + \Delta E_{elst} + \Delta E_{oi} + \Delta E_{disp} \qquad [1]$$

where ΔE_{Pauli} represents the Pauli repulsion interaction between occupied orbitals on the two fragments, ΔE_{elst} is the quasiclassical electrostatic interaction between the unperturbed charge distribution of the fragments at their final positions, ΔE_{disp} takes into account the dispersion contribution and ΔE_{oi} is the orbital interaction, which arises from the orbital relaxation and the orbital mixing between the fragments, and accounts for electron pair bonding, charge transfer, and polarization.

- **Natural Orbitals for Chemical Valence (NOCV) and Charge Displacement (CD) analysis**

The Natural Orbitals for Chemical Valence (NOCV)[4,5] is a suitable approach for the description of chemical bonding and is based on the rearrangement of the electron density occurring when a chemical bond is formed. In general, arrangement can be expressed as electron density difference between the formed adduct (AB) and sum of the densities of the two non-interacting fragments (A and B) frozen in the geometries they have in the adduct.

This deformation density can be brought into diagonal contributions in terms of NOCVs. In the NOCV scheme, the charge rearrangement taking place upon bond formation is obtained from the occupied orbitals of the two fragments suitably orthogonalized to each other and renormalized (*promolecule*). The resulting electron density rearrangement ($\Delta\rho$) can be expressed in terms of NOCV pairs which are defined as the eigenfunctions of the so-called "valence operator"[6–8] as follows:

$$\Delta\rho = \sum_k v_k(|\phi_{+\mathrm{k}}|^2 - |\phi_{-\mathrm{k}}|^2) = \sum_k \Delta\,\rho'_k \qquad [2]$$

where ϕ_{+k} and ϕ_{-k} are the NOCV pairs orbitals and $v_{\pm k}$ are the corresponding eigenvalues. When the adduct is formed from the promolecule, a fraction v_k of electrons is transferred from the ϕ_{-k} to the ϕ_{-k} orbital, which are envisaged as donor and acceptor orbitals, respectively. For the sake of interpretation, a population analysis can also be performed in order to single out, for ϕ_{-k} and ϕ_{+k} orbitals, which molecular orbitals (MOs) of the two constituting fragments contribute to the interaction (with a resulting associated coefficient accounting for the magnitude of the contribution).

The NOCV scheme can be coupled with the framework of the Charge Displacement (CD) analysis. The CD analysis allows to quantify the amount of electronic charge that is transferred between the two fragments upon the formation of the A-B bond. The Charge Displacement function (Δq) is defined as the partial progressive integration on a suitable z-axis of the deformation density $\Delta\rho$:[9]

$$\Delta q(z) = \int_{-\infty}^{z} dz' \int_{-\infty}^{+\infty}\int_{-\infty}^{+\infty} \Delta\,\rho(x,y,z')dxdy \qquad [3]$$

The CD function, Δq(z), quantifies at each point of the bond axis the exact amount of electron charge that, upon formation of the bond, is transferred from the right to the left across a plane perpendicular to the bond axis through z.

The CD and NOCV frameworks can be coupled in the CD-NOCV scheme.[10] In the latter, the density rearrangement due to the bond formation between two fragments, ($\Delta\rho$), can be partitioned in different NOCV deformation densities ($\Delta\rho_k$) and therefore one is able to quantify the charge transfer (CT) associated to each different component. It must be noted that only few of the NOCV pairs contributes to the chemical bond. Therefore, when the CD-NOCV analysis is carried out,

usually only the first $\Delta\rho_k$ components are investigated in order to understand which significant chemical contribution to the bond they represent.

In equation [3], the integration axis is usually conveniently chosen as the bond axis between the two fragments constituting the adduct and usually we choose to evaluate the charge transfer between A and B by taking the CD value at the "isodensity boundary", i.e. the z-point where equally valued isodensity surfaces of the isolated fragments become tangent.[9]

In this case, we apply this scheme to the transition states TS_{Si}^{Zn2} with $[Si^{II}(^{Dipp}BDI\text{-}H)]$ and $[Cp^{*}ZnZnCp^{*}]$ as fragments; TS_{Al}^{Zn2} with $[Al^{I}(NacNac)]$ and $[Cp^{*}ZnZnCp^{*}]$ as fragments; TS_{Si}^{H2} with $[Si^{II}(^{Dipp}BDI\text{-}H)]$ and $[H_2]$ as fragments; and TS_{Al}^{H2} with $[Al^{I}(NacNac)]$ and $[H_2]$ as fragments. However, the standard approach is complicated, since the two fragments display multiple interactions with multiple atomic centers and thus it is clearly impossible to define a unique bond axis and it is very hard to rely on the isodensity boundary for the estimation of the charge transfer. In order to avoid any ambiguity in the definition of the z-axis, we recall an approach that may be useful for evaluating the charge transferred between the two fragments at the transition state.[11]

Within this approach, the electron density rearrangement ($\Delta\rho$), which typically shows charge accumulation regions (positive values) and charge depletion regions (negative values), defines two different positive functions, $\Delta\rho^{+}$ and $\Delta\rho^{-}$, each equal to the magnitude of the appropriate portion, *i.e.*:

$$\Delta\rho^{+/-}(r) = max[\pm\Delta\rho(r), 0] \qquad [4]$$

so that

$$\Delta\rho(r) = \Delta\rho^{+}(r) - \Delta\rho^{-}(r) \qquad [5]$$

By defining two arbitrary regions that are associated with the interacting fragments, we can evaluate the charge transfer as follows:

$$CT = \int_A \Delta\,\rho(r)dr = -\int_B \Delta\,\rho(r)dr \qquad [6]$$

By combining Eqs. [5] and [6], CT can also be expressed as:

$$CT = \int_A \Delta\, \rho^+(r)dr - \int_A \Delta\, \rho^-(r)dr = -\int_B \Delta\, \rho^+(r)dr + \int_B \Delta\, \rho^-(r) \quad [7]$$

Ultimately, this approach can also be expressed in the CD-NOCV framework. By combining Equations [2] and [6], we can use to this approach for calculating the charge transfer associated to each NOCV deformation density as follows:

$$CT_k = \int_A \Delta\, \rho_k(r)dr = -\int_B \Delta\, \rho_k(r)dr \quad [7]$$

Despite the spatial regions associated to the two interacting fragments being defined arbitrarily, this approach is particularly suitable for the analysis of the interaction between the four considered couples of fragments in TS_{Si}^{Zn2}, TS_{Al}^{Zn2}, TS_{Si}^{H2} and TS_{Al}^{H2}, being the two fragments well-separated in space in all cases, therefore removing any ambiguity in the fragmentation choice.

- **Effective Fragment Orbitals and Effective Oxidation State analyses**

In 1995, Mayer introduced the concept of effective atomic orbitals (eff-AOs),[12] providing an alternative to the traditional description based on atomic orbitals. These-eff-AOs are constructed for each atom by diagonalizing the atom's net density, yielding distorted orbitals that retain the shape of atomic orbitals. Molecular fragments can be defined for practical application, and Mayer's eff-AOs can be generalized to effective fragment orbitals (EFOs).[13] To generate these eff-AOs, let us consider a spin-unrestricted Single-determinant wavefunction built from singly n_σ occupied molecular orbitals, $\{\phi_i^\sigma(r)\}_{i=1,n_\sigma}$, where $\sigma = \alpha, \beta$. In a general real-space formalism, for each atom A, we can define the intra-atomic part of every MO as

$$\phi_i^{A,\sigma}(\boldsymbol{r}) = \mathrm{w}_A(\boldsymbol{r})\phi_i^\sigma(\boldsymbol{r}), \quad [8]$$

where $\mathrm{w}_A(\boldsymbol{r})$ is a non-negative, continuous atomic weight function fulfilling $\sum_A \mathrm{w}_A(\boldsymbol{r}) = 1$, which defines the fuzzy domain of atom A in the molecule.

For each spin case, the Hermitian matrix $Q_{ij}^{A,\sigma}$ can be built with the elements

$$Q_{ij}^{A,\sigma} = \int \phi_i^{A,\sigma^*}(\boldsymbol{r})\phi_j^{A,\sigma}(\boldsymbol{r})d\boldsymbol{r} = \int w_A^*(\boldsymbol{r})\phi_i^{\sigma^*}(\boldsymbol{r})\phi_j^\sigma(\boldsymbol{r})w_A(\boldsymbol{r})\, d\boldsymbol{r}, \quad [9]$$

and it is essentially the *net* atomic overlap matrix in the basis of the MOs. In order to determine the eff-AOs corresponding to atom A, diagonalization of the $Q^{A,\sigma}$ by the unitary matrix $U^{A,\sigma}$ is necessary

$$\boldsymbol{U}^{A,\sigma\dagger}\boldsymbol{Q}^{A,\sigma}\boldsymbol{U}^{A,\sigma} = diag\{\lambda_i^{A,\sigma}\}. \quad [10]$$

We obtain n_A eff-AOs as linear combinations of the *intraatomic* parts of the MOs as

$$\varphi_i^{A,\sigma}(r) = \frac{1}{\sqrt{\lambda_i^{A,\sigma}}}\sum_{\mu=1}^{n_\sigma} U_{\mu i}^{A,\sigma}\phi_\mu^{A,\sigma}(\boldsymbol{r}) \quad i = 1,2,\dots,n_\sigma^A. \quad [11]$$

where n_σ^A is the number of non-zero eigenvalues $\lambda_i^{A,\sigma}$. The occupation numbers of each eff-AO are given by the eigenvalues $0 \leq \lambda_i^{A,\sigma} \leq 1$. The shape and occupation numbers of the eff-AOs accurately reflect the core and valence shells of the atoms: those with occupation numbers near 1 represent core orbitals or lone pairs, while those with smaller yet notable occupations are associated with the atomic orbitals involved in bonding. The remaining eff-AOs have marginal occupations and lack any significant chemical relevance. In case molecular fragments are defined, we obtain EFOs by using fragment weight functions of the following form in Eq. 8

$$w_K(\boldsymbol{r}) = \sum_{i\epsilon K} w_i(\boldsymbol{r}), \quad [12]$$

where the sum runs for all atoms of molecular fragment *K*.

The so-called effective oxidation state (EOS) analysis,[13] relies on the occupation number of Mayer's eff-AOs obtained for all atoms or molecular fragments defined, and goes as follows. First, the EFOs are obtained for all atoms/fragments considered in the compound and sorted by decreasing occupation number. Then, the total number of electrons is distributed among the EFOs of different fragments by comparing their occupation numbers (alpha and beta separately in open-shell systems). This procedure yields an effective electronic configuration for each fragment, from which the difference in its atomic number determines the effective oxidation state (EOS). It is important to note that the occupation numbers of the EFOs are not merely rounded to the nearest integer. Instead, they are sorted in decreasing order for each spin case. The first n_σ EFOs are considered occupied, being n_σ the total number of electron for the spin case σ.

Additionally, the difference between the occupation number of the last occupied (LO) and first unoccupied (FU) EFOs provide a quantitative measure of the reliability of the assignment. The larger this difference, the better the current electron distribution can be described within a discrete

ionic model. Accordingly, the EOS analysis provides, together with the OS assignment, an associated reliability index, $R_\sigma(\%)$ for each spin component, defined as

$$R_\sigma(\%) = 100 \cdot \min(1, \max(0, \lambda_{LO}^\sigma - \lambda_{FU}^\sigma + 1/2)). \quad [13]$$

The R index reaches 100% when the occupation difference between the frontiers EFOs exceeds half an electron. In contrast, the least reliable scenario corresponds to the case in which two or more frontiers EFOs belonging to different fragments are degenerate in occupation, yielding R(%)=50.

Optimized stationary points

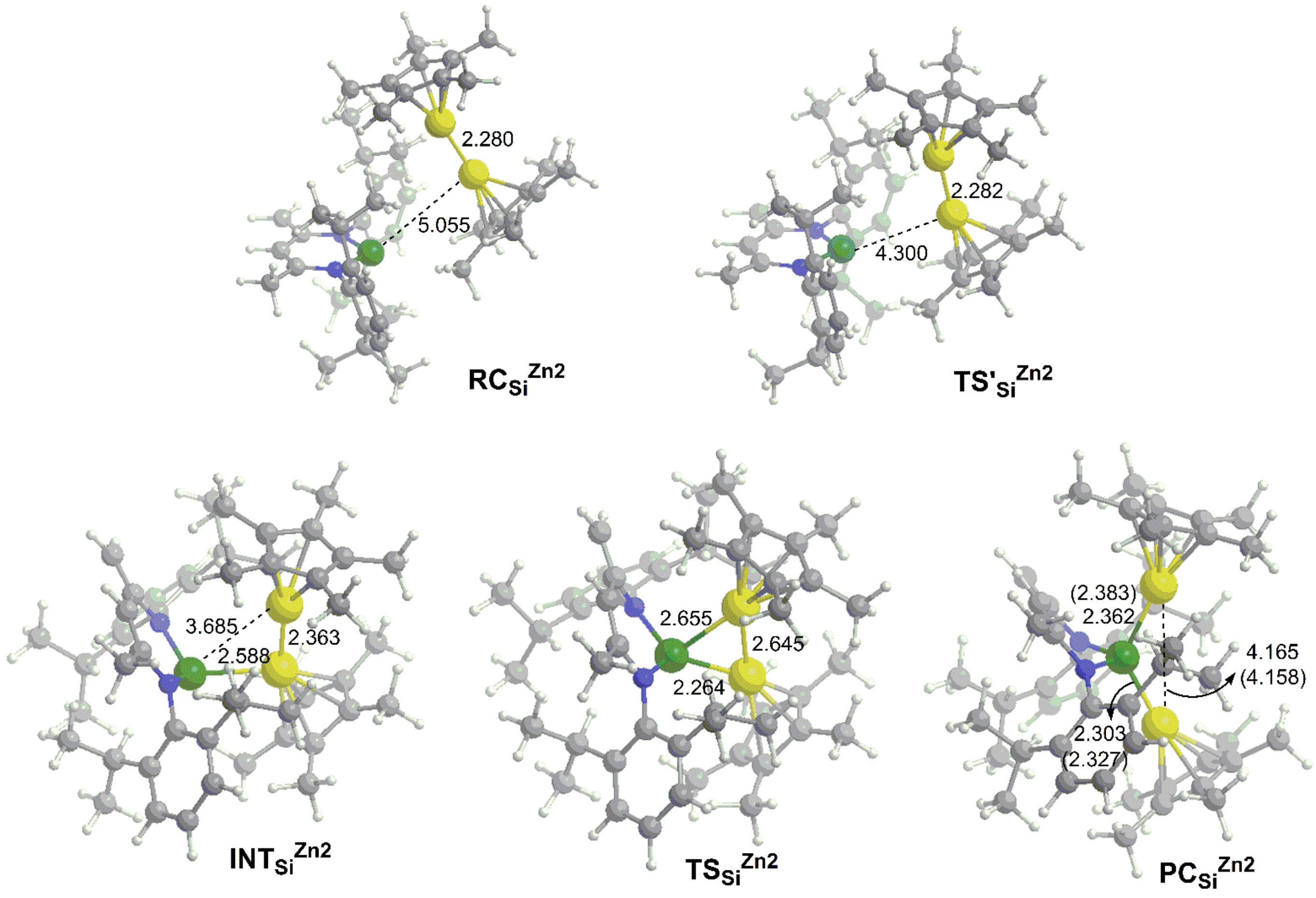

Figure S1. Optimized structures of all the stationary points along the profile for the addition of Cp*ZnZnCp* to **1Si**. Main geometrical parameters are reported (bond in Å). Experimental solid-state values are shown in parenthesis.

The imaginary frequency of TS'_{Si}^{Zn2} is very small (-23.0 cm^{-1}), indicating a very flat potential energy surface associated to a complex vibrational motion mainly involving bending of one isopropyl group of the Dipp moiety at the Cp*ZnZnCp* approach. This transition state highlights the importance of including dispersion corrections in the calculations, which were not included in Ref. [14] to the best of our knowledge, thus hindering its identification. The TS_{Si}^{Zn2} instead possesses one larger imaginary frequency (-130.7 cm^{-1}) which is associated to a vibrational motion engaging

the Si and the two Zn atoms. A concerted transition state, where the Zn-Zn bond is breaking while the two Si-Zn bonds are forming, is clearly suggested.

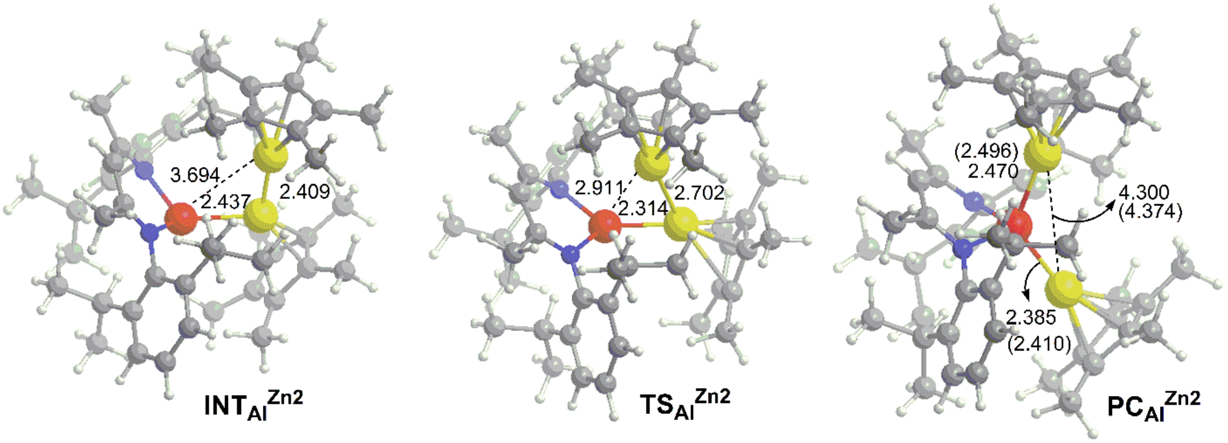

Figure S2. Optimized structures of all the stationary points along the profile for the addition of Cp*ZnZnCp* to **1Al**. Main geometrical parameters are reported (bond in Å). Experimental solid-state values are shown in parenthesis.

The TS_{Al}^{Zn2} possesses one imaginary frequency (-111.9 cm^{-1}) which is associated to a vibrational motion involving the Al and the two Zn atoms. A concerted transition state, where the Zn-Zn bond is breaking while the two Al-Zn bonds are forming, is clearly suggested.

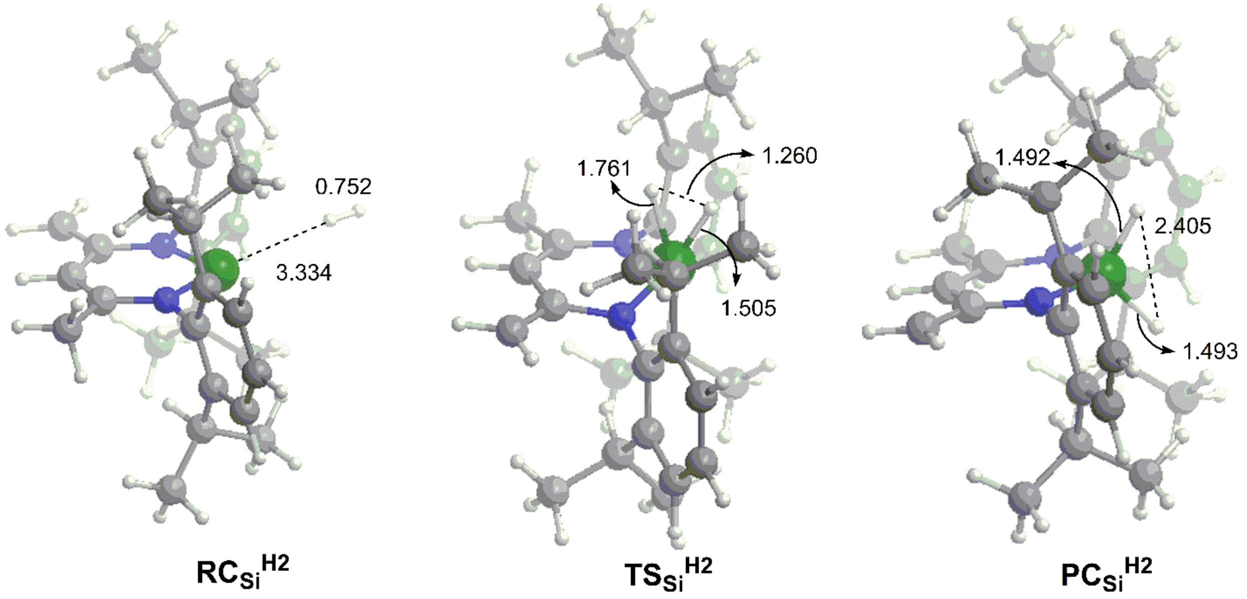

Figure S3. Optimized structures of all the stationary points along the profile for the addition of H_2 to **1Si**. Main geometrical parameters are reported (bond in Å).

The TS_{Si}^{H2} possesses one large imaginary frequency (-1245.4 cm^{-1}) which is associated to a vibrational motion involving the Si and the two H atoms. A concerted transition state, where the H-H bond is breaking while the two Si-H bonds are forming, is clearly suggested.

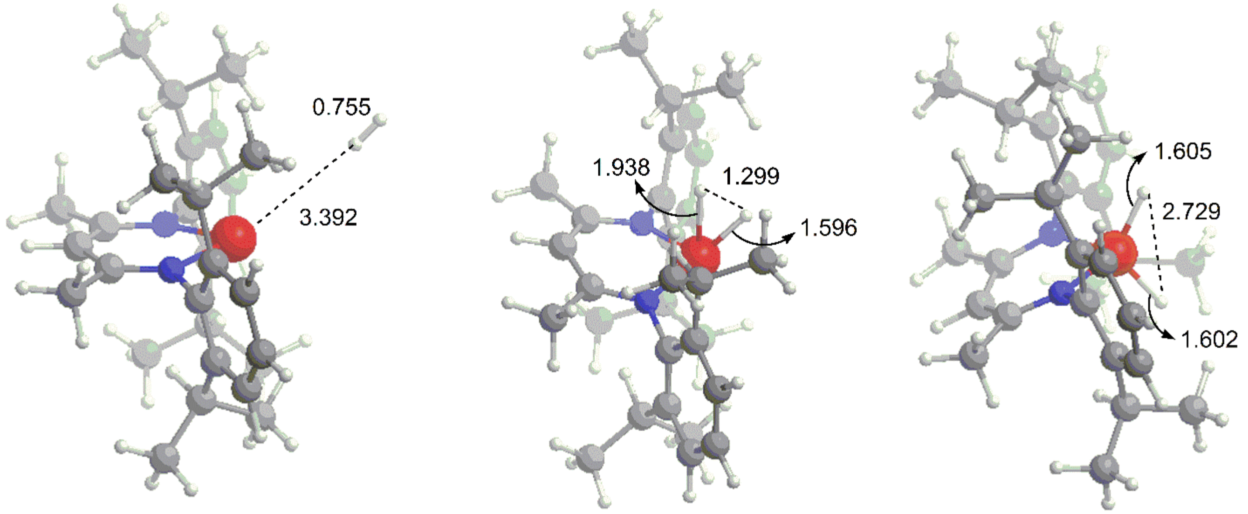

RC_{Al}^{H2} TS_{Al}^{H2} PC_{Al}^{H2}

Figure S4. Optimized structures of all the stationary points along the profile for the addition of H_2 to **1Al**. Main geometrical parameters are reported (bond in Å).

The TS_{Al}^{H2} possesses one large imaginary frequency (-1186.5 cm^{-1}) which is associated to a vibrational motion involving the Al and the two H atoms. A concerted transition state, where the H-H bond is breaking while the two Al-H bonds are forming, is clearly suggested.

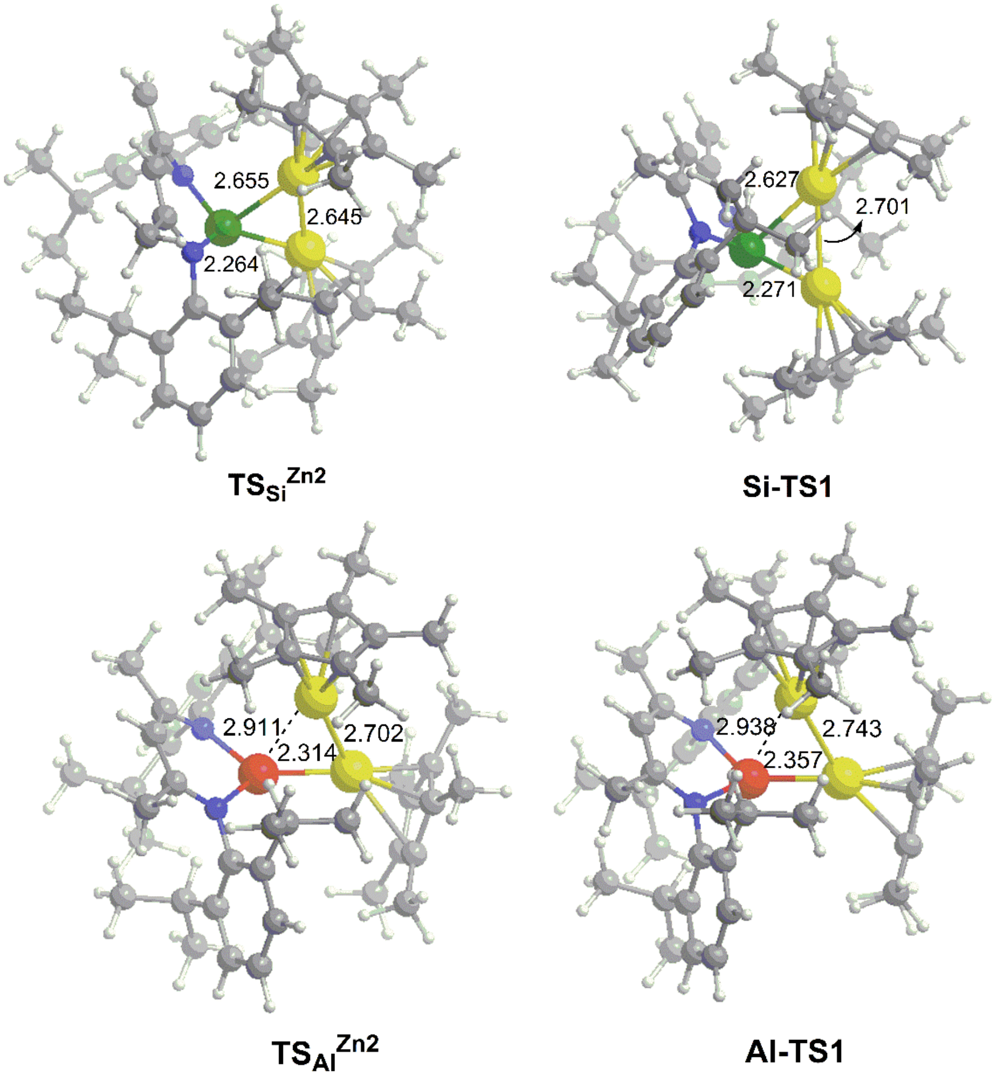

Figure S5. Comparison between the optimized structures of the transition states TS$_{Si}^{Zn2}$ and TS$_{Al}^{Zn2}$ calculated in this work and those of the corresponding transition states Si-TS1and Al-TS1 calculated in Ref. [14] with a different computational set up. Main geometrical parameters are reported (bond in Å).

Figure S5 shows a comparison between the optimized structures of the transition states TS$_{Si}^{Zn2}$ and TS$_{Al}^{Zn2}$ calculated in this work and those of the corresponding transition states Si-TS1and Al-TS1 calculated in Ref. [14] with a different computational set up. We observe a good agreement between the structures (i.e., in all cases three-center transition states have been calculated), although some differences can be observed in the bond lengths, consistent with the different computational protocol (G09: M06L/def2TZVPP/PCM(benzene)//M06L/6-31G**/6-311+G*/SDDAll (Al, Zn)).

System	ΔE_{int}	ΔE_{Pauli}	ΔE_{elst}	ΔE_{orb}	ΔE_{disp}
TS_{Al}^{Zn2}	-60.65	294.44	-179.04	-127.44	-23.60
TS_{Al}^{H2}	-25.24	184.54	-68.69	-139.21	-2.18
TS_{Si}^{Zn2}	-48.49	293.50	-177.82	-137.19	-27.43
TS_{Si}^{H2}	-16.15	245.07	-92.74	-167.15	-1.32

Table S1. Energy Decomposition Analysis of the interaction between H_2/Cp*ZnZnCp* and **1Al** and **1Si** at TS_E^{H2} and TS_E^{Zn2}. The overall interaction energy (ΔE_{int}), the Pauli repulsion (ΔE_{Pauli}), electrostatic (ΔE_{elst}), orbital interaction (ΔE_{orb}), and dispersion (ΔE_{disp}) contributions are reported in kcal/mol.

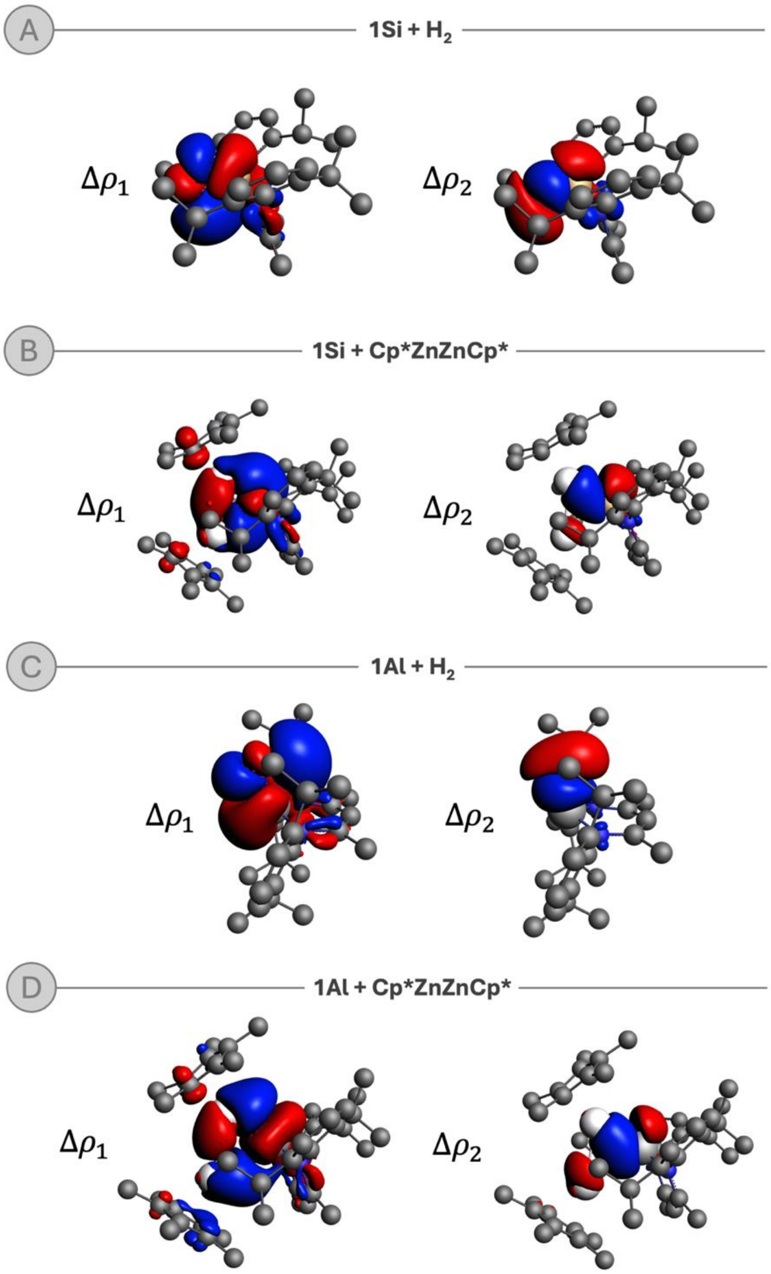

Figure S6. Dominant NOCV deformation densities ($\Delta\rho_1$ and $\Delta\rho_2$) describing the interaction between **1Si** and **1Al** with H_2 (A and C, respectively) and Cp*ZnZnCp* (B and D, respectively). Red (blue) regions indicate electron density depletion (accumulation). The ligand hydrogen atoms have been removed for clarity. The isovalue for all isosurfaces is 1 me/a_0^3.

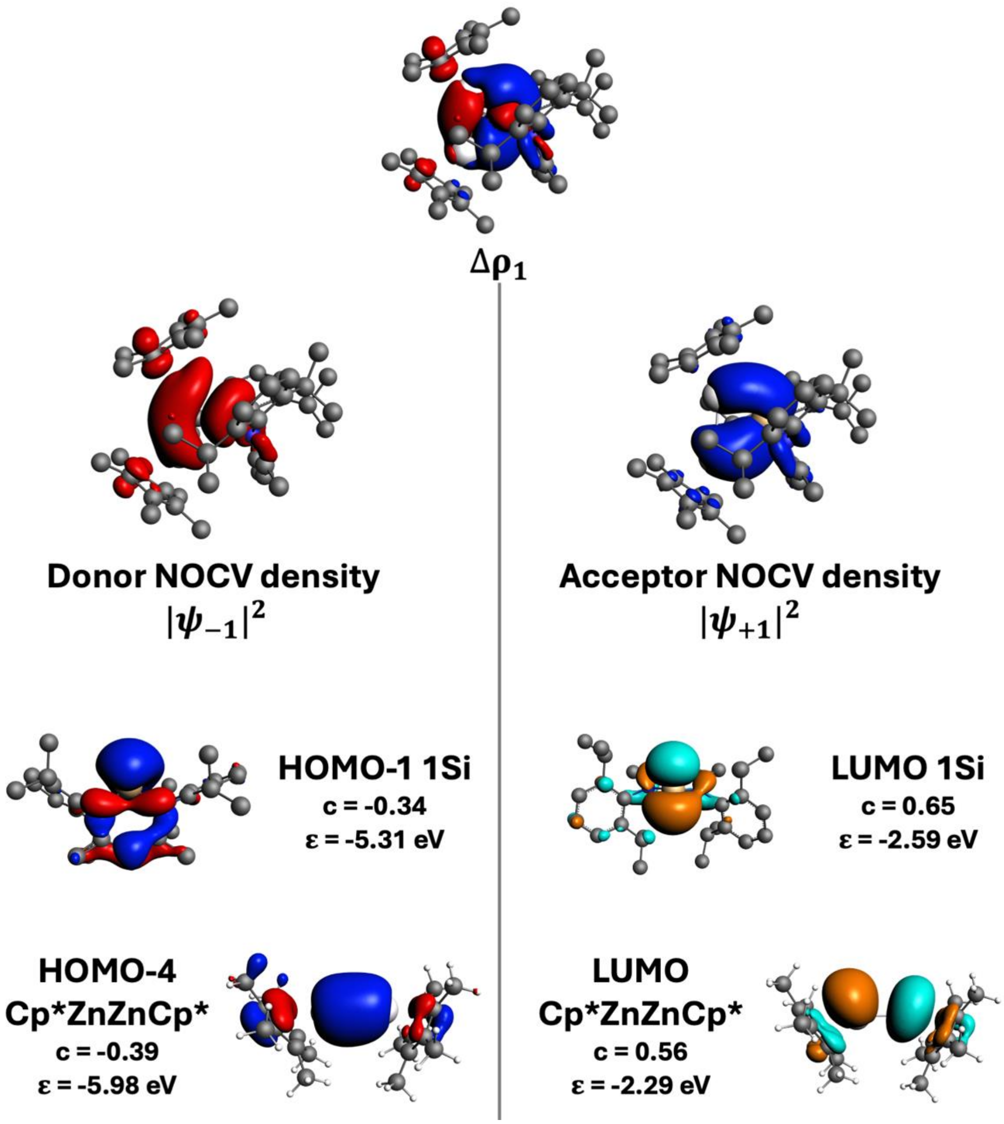

Figure S7. Breakdown of the donor ($|\psi_{-1}|^2$) and acceptor ($||\psi_{+1}|^2$) NOCV densities that are associated with the deformation density $\Delta\rho_1$ in the transition state TS_{Si}^{Zn2} into the most important MOs of the fragments frozen at their TS geometry. The mixing coefficients and the in-adduct energies are reported for each MO.

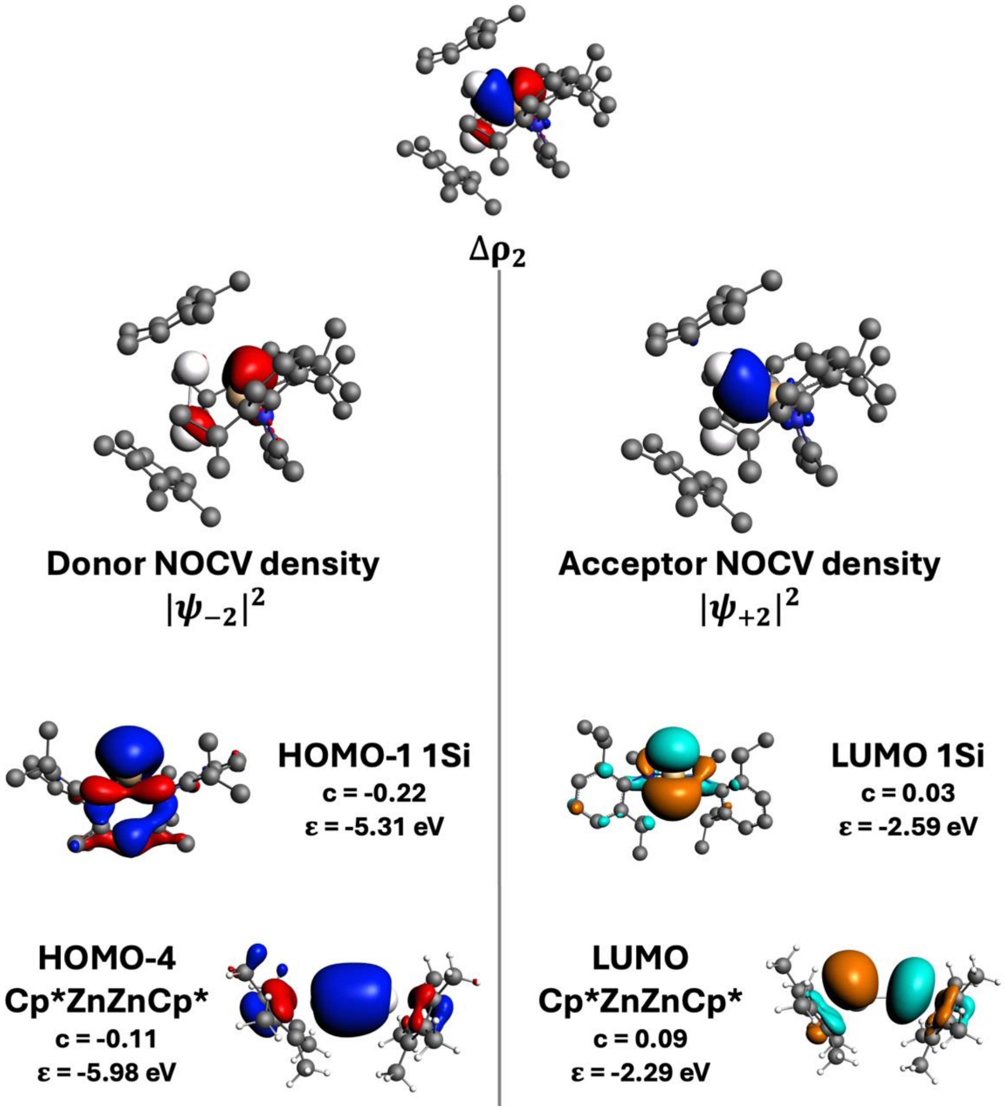

Figure S8. Breakdown of the donor ($|\psi_{-2}|^2$) and acceptor ($||\psi_{+2}|^2$) NOCV densities that are associated with the deformation density $\Delta\rho_2$ in the transition state TS_{Si}^{Zn2} into the most important MOs of the fragments frozen at their TS geometry. The mixing coefficients and the in-adduct energies are reported for each MO.

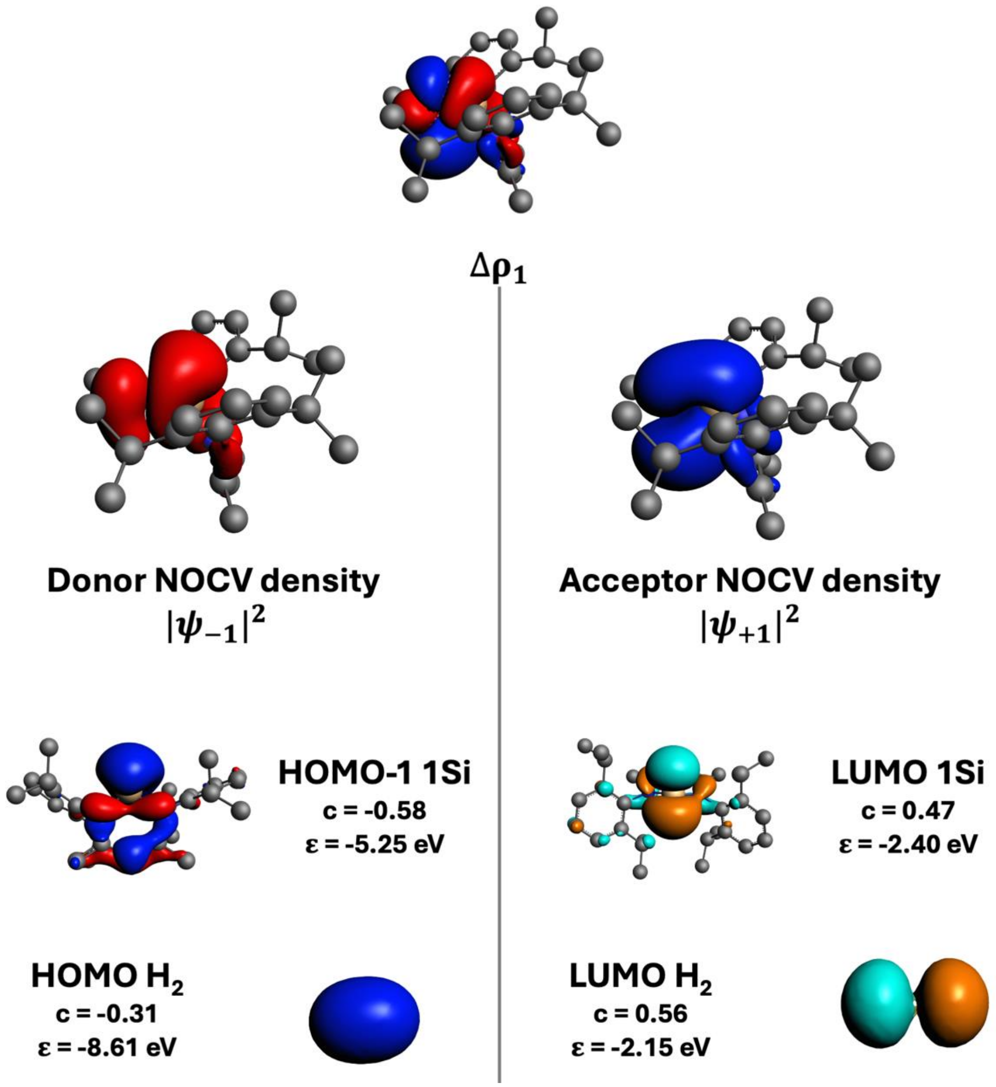

Figure S9. Breakdown of the donor ($|\psi_{-1}|^2$) and acceptor ($||\psi_{+1}|^2$) NOCV densities that are associated with the deformation density $\Delta\rho_1$ in the transition state TS_{Si}^{H2} into the most important MOs of the fragments frozen at their TS geometry. The mixing coefficients and the in-adduct energies are reported for each MO.

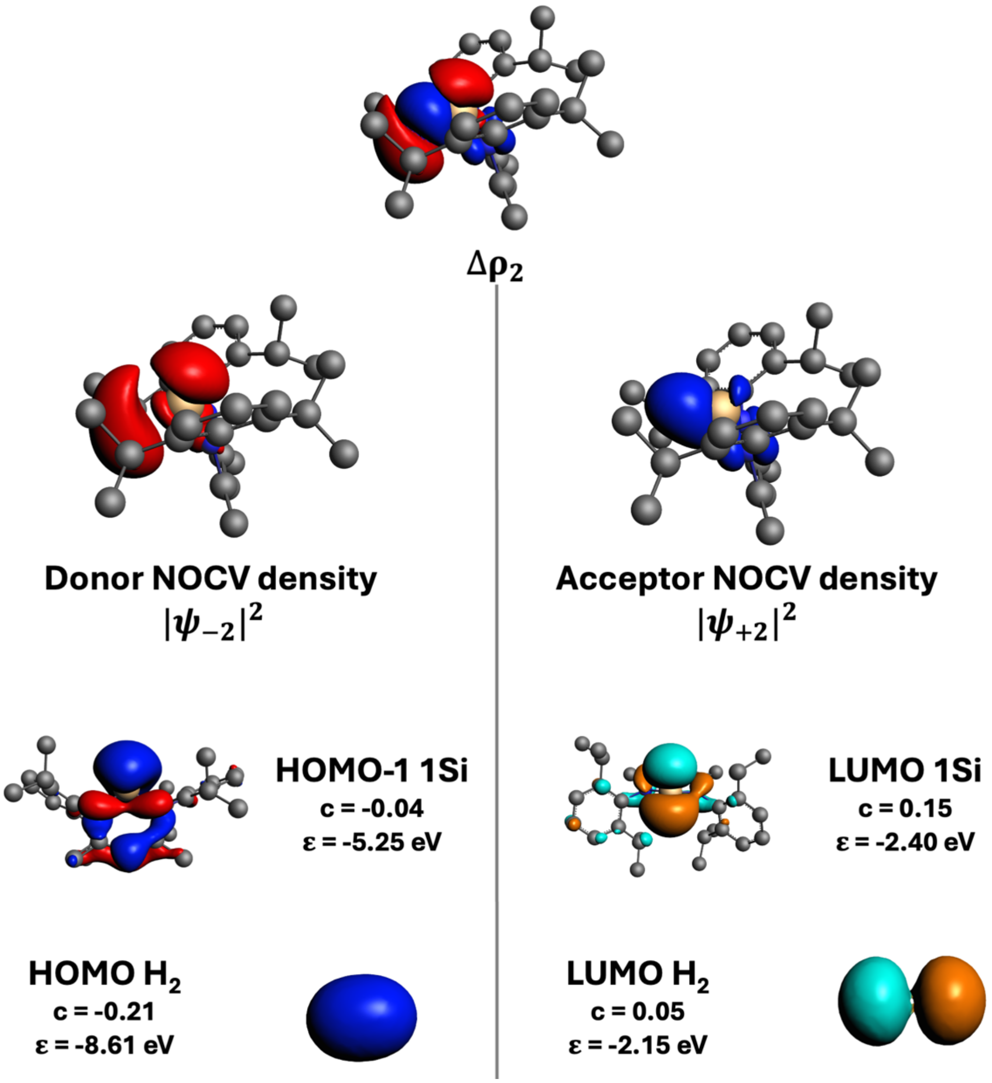

Figure S10. Breakdown of the donor ($|\psi_{-2}|^2$) and acceptor ($||\psi_{+2}|^2$) NOCV densities that are associated with the deformation density $\Delta\rho_2$ in the transition state TS_{Si}^{H2} into the most important MOs of the fragments frozen at their TS geometry. The mixing coefficients and the in-adduct energies are reported for each MO.

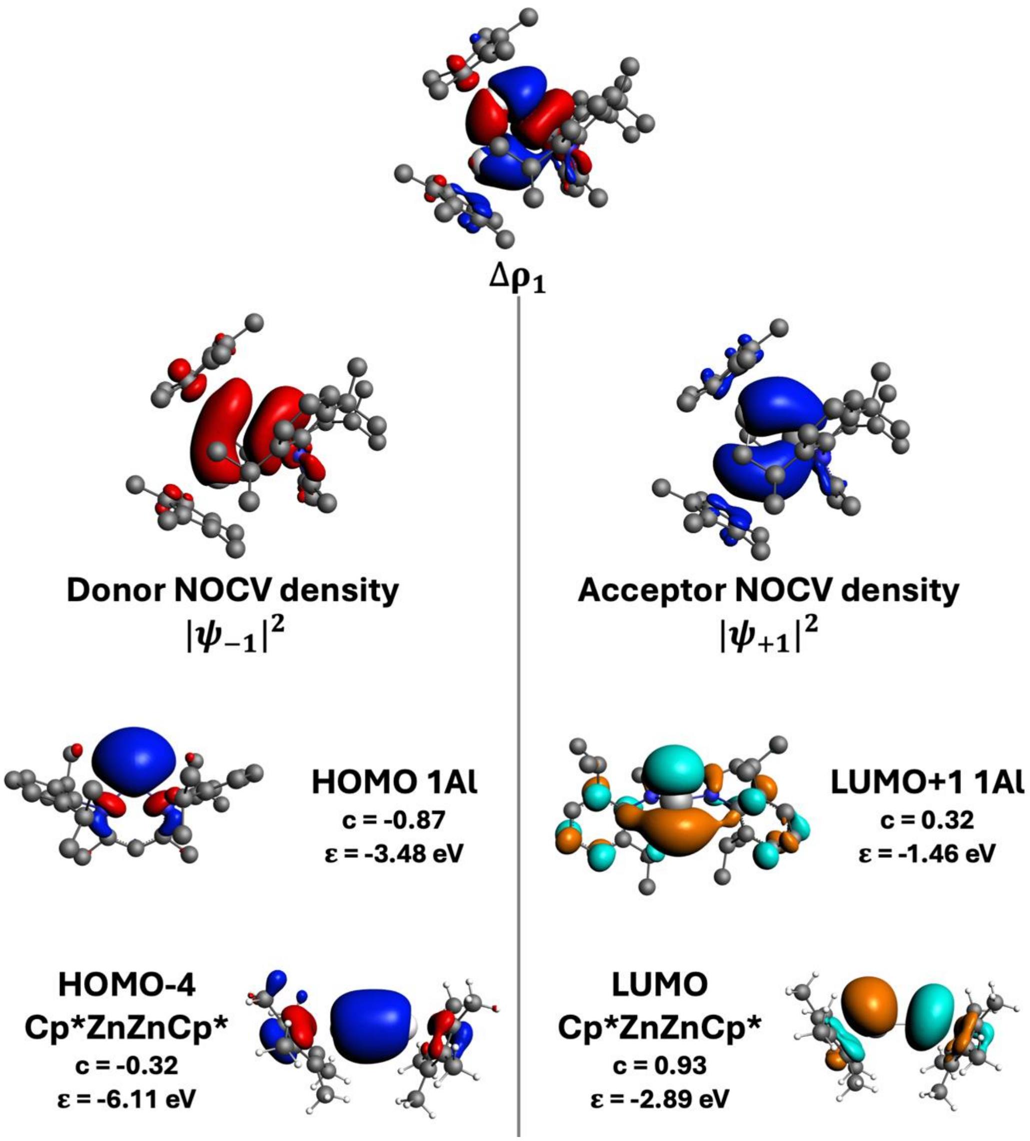

Figure S11. Breakdown of the donor ($|\psi_{-1}|^2$) and acceptor ($||\psi_{+1}|^2$) NOCV densities that are associated with the deformation density $\Delta\rho_1$ in the transition state TS_{Al}^{Zn2} into the most important MOs of the fragments frozen at their TS geometry. The mixing coefficients and the in-adduct energies are reported for each MO.

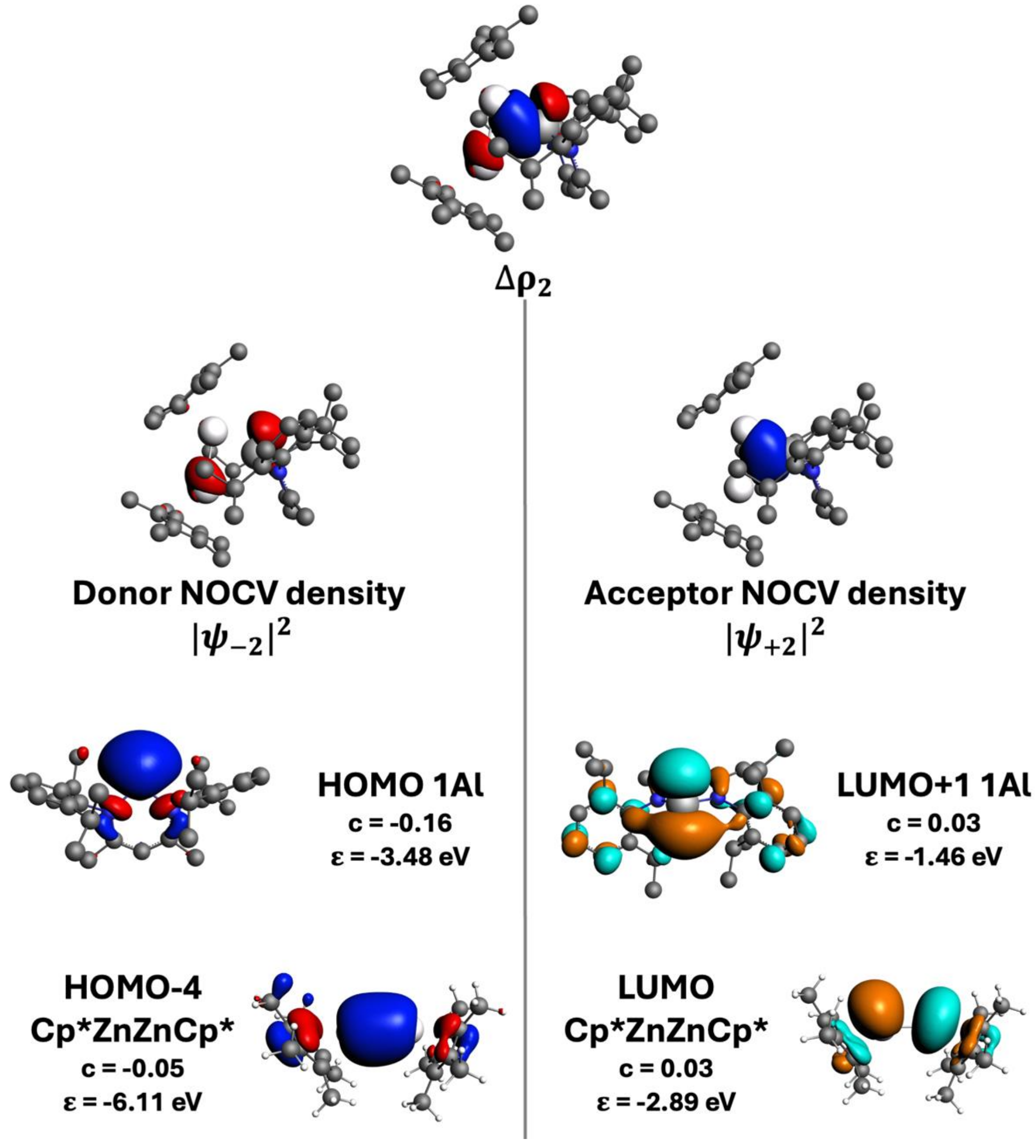

Figure S12. Breakdown of the donor ($|\psi_{-2}|^2$) and acceptor ($||\psi_{+2}|^2$) NOCV densities that are associated with the deformation density $\Delta\rho_2$ in the transition state TS_{Al}^{Zn2} into the most important MOs of the fragments frozen at their TS geometry. The mixing coefficients and the in-adduct energies are reported for each MO.

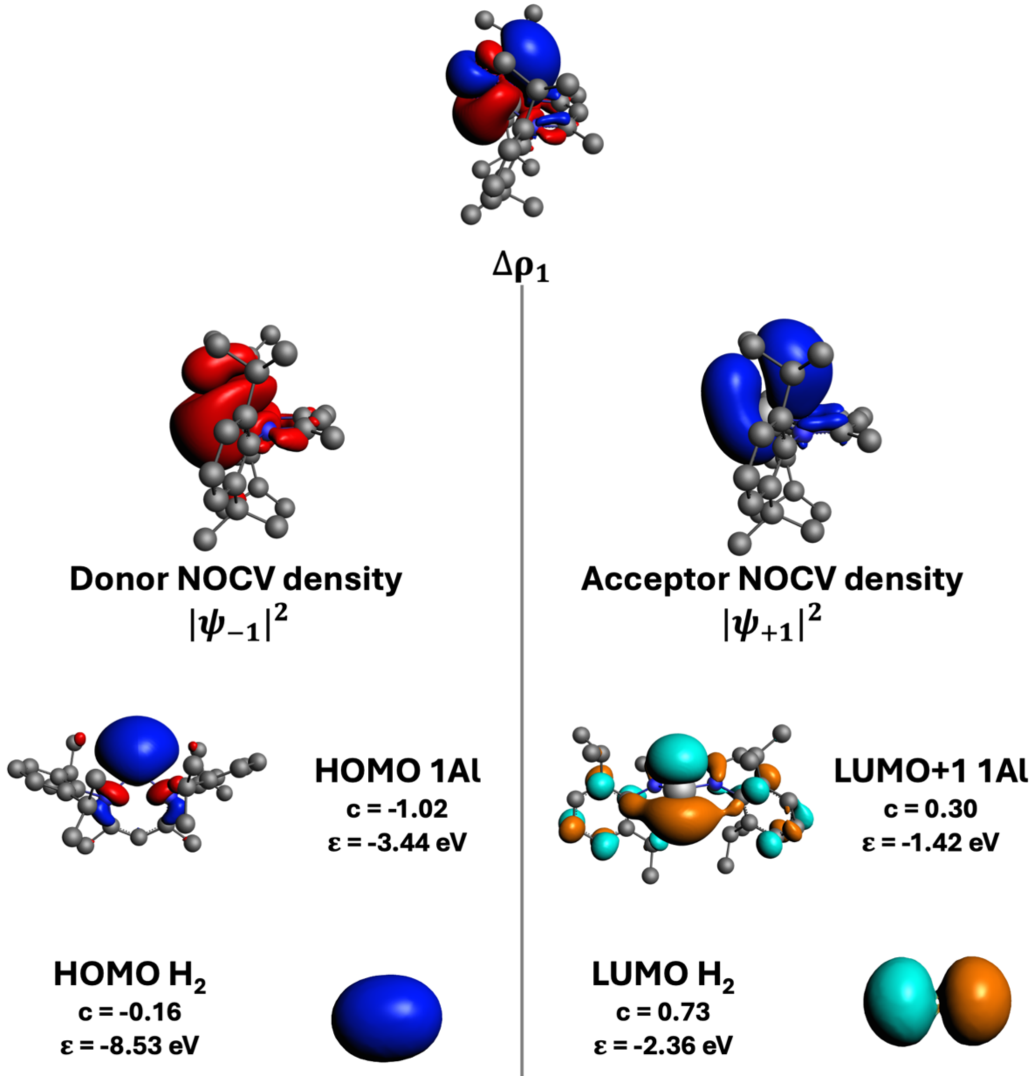

Figure S13. Breakdown of the donor ($|\psi_{-1}|^2$) and acceptor ($||\psi_{+1}|^2$) NOCV densities that are associated with the deformation density $\Delta\rho_1$ in the transition state TS_{Al}^{H2} into the most important MOs of the fragments frozen at their TS geometry. The mixing coefficients and the in-adduct energies are reported for each MO.

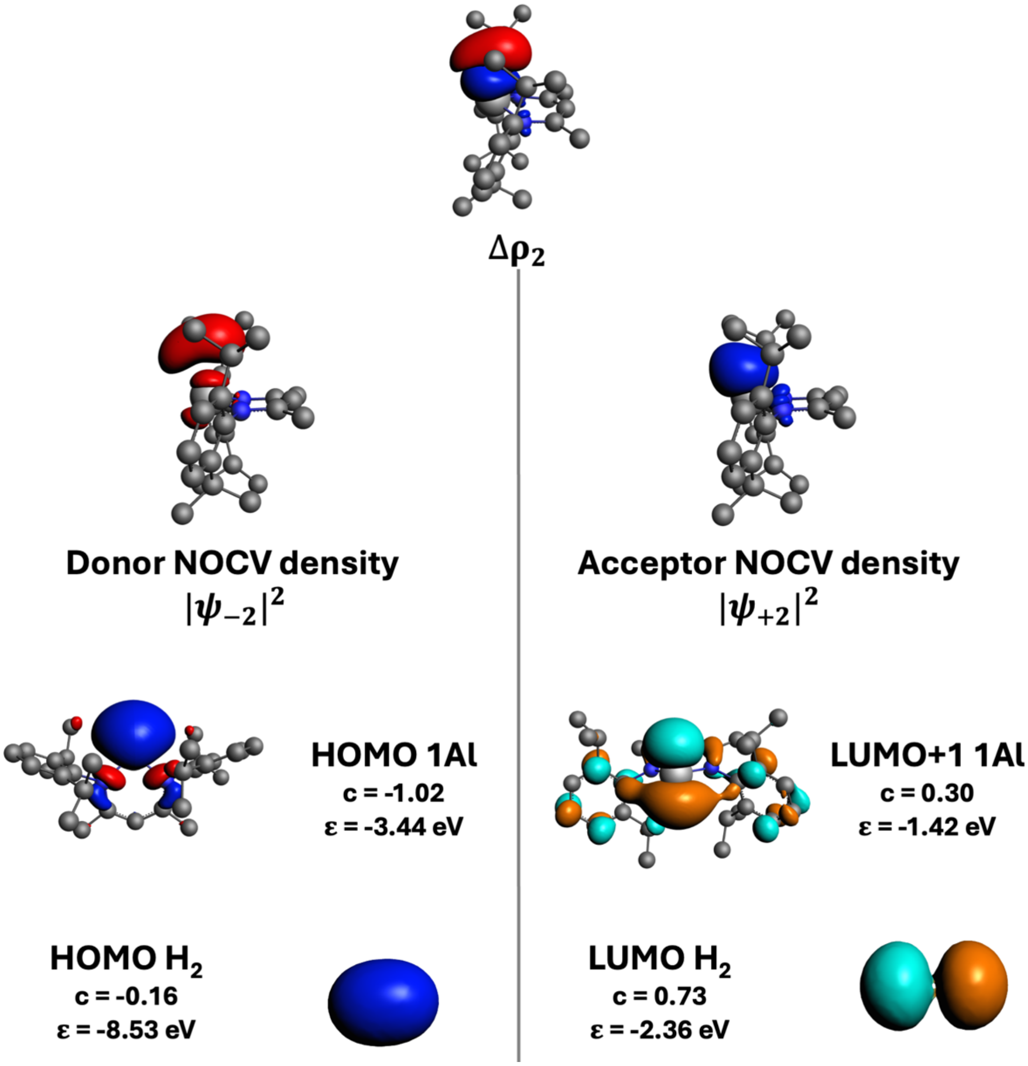

Figure S14. Breakdown of the donor ($|\psi_{-2}|^2$) and acceptor ($||\psi_{+2}|^2$) NOCV densities that are associated with the deformation density $\Delta\rho_2$ in the transition state TS_{Al}^{H2} into the most important MOs of the fragments frozen at their TS geometry. The mixing coefficients and the in-adduct energies are reported for each MO.

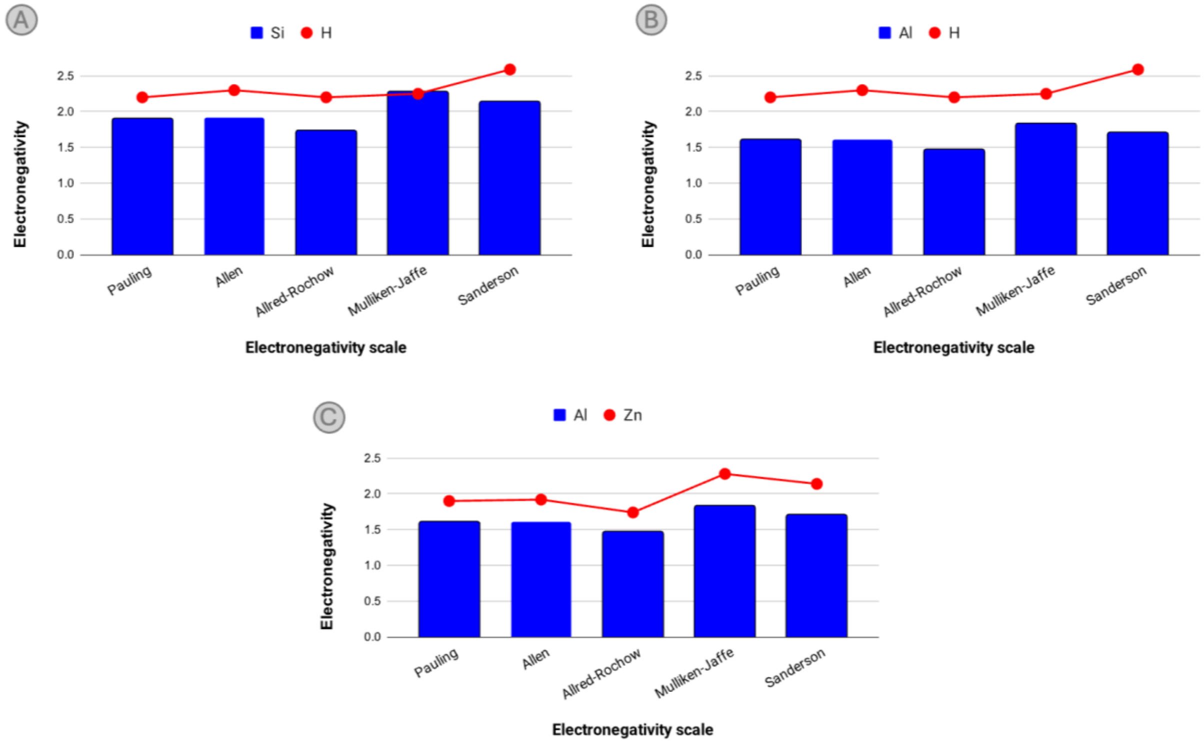

Figure S15. Comparative analysis of the atomic electronegativities of Si and H (A), Al and H (B) and Al and Zn (C) across different scales.

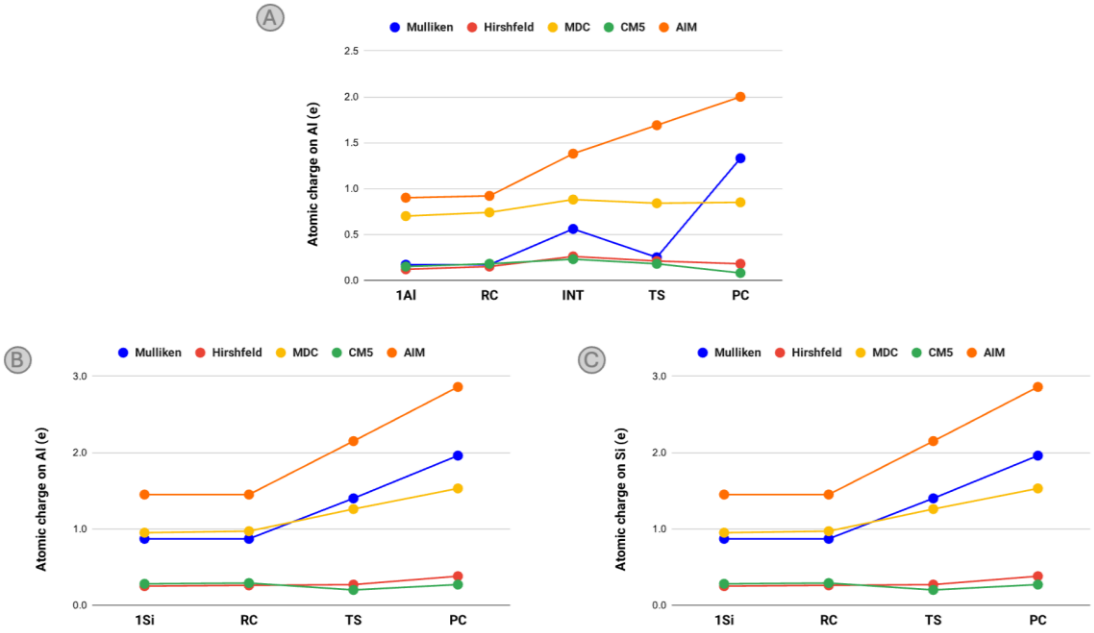

Figure S16. Computed atomic charges on Al and Si along the path for the reaction of **1Al** with Cp*ZnZnCp* (A) and H_2 (B) and of **1Si** with H_2 (C).

Effective Oxidation State (EOS) analysis

To monitor oxidation-state variations along the reaction pathway, we first applied EOS analysis to the reference **1Al/1Si** compounds. For **1Al**, the system contains 121 alpha and 121 beta electrons to be distributed among the EFOs. As the wavefunction is restricted, the analysis was performed only for the alpha part, since the beta manifold yields identical results. Table S2 reports the sorted EFO occupation numbers for each fragment (main group element and ligand scaffold), with highly occupied EFOs highlighted in bold. It is shown that 6 alpha EFOs are occupied for Al and 115 alpha EFOs are occupied for the NacNac ligand. Hence, a total of 12 and 230 electrons are awarded to Al and NacNac, respectively. This electron distribution is consistent with a formal Al (+1) and NacNac ligand (-1) OS assignment. An analogous analysis for **1S**i yields Si (+2) and ^{Dipp}BDI-H ligand (-2) OSs, confirming that the method captures the expected reference OSs. Having validated the EOS analysis for the reference compounds, we next examined the addition step. The resulting oxidation states of the main-group center and ligands, along with the corresponding R(%) indices, are summarized in Table S3. Notably, EOS analysis consistently assigns an OS of -1 to the NacNac ligand in the PCAl systems and -2 in the DippBDI-H ligand in the PCSi analogues, with EFO occupation patterns closely resembling those of the **1Al/1Si** reference complexes. This observation indicates that the addition step does not significantly alter the electronic structure of the ligand scaffold, as expected.

Focusing on dihydrogen addition, the central main-group elements are fully oxidized, yielding Al(+3) and Si(+4) OSs. Each hydrogen ligand is assigned an anionic (-1) character, consistent with an oxidative addition mechanism as discussed above. Beyond OS assignment, it is interesting to note that the last occupied EFO is localized on the H ligand, corresponding to the σ lone pair, whereas the first unoccupied EFO is centered on E and exhibits s-type character. The clear separation between these frontier EFOs leads to an unambiguous assignment, with $R > 95$.

Applying EOS analysis to PC_{Al}^{Zn2} reveals a similarly clear picture, with Al(+3) and ZnCp*(-1) OS assignments, in close analogy to PC_{Al}^{H2}. The occupation of the σ-type EFO of the ZnCp* ligand slightly decreases from 0.77 in the H ligand to 0.67, while the occupation of the s-type EFO on Al increases from 0.23 to 0.30. Together, these changes reduce the R(%) value, yet the oxidative character of the addition remains unambiguous. In contrast, PC_{Si}^{Zn2} exhibits a markedly different electronic situation. The occupation of the s-type EFO on Si increases markedly, reaching 0.57. In parallel, the occupation of the σ-type EFO associated with the two ZnCp* ligands decreases to

0.45. As a consequence, EOS analysis assigns the s-type EFO as occupied, resulting in an OS of +2 for the Si center. The remaining two σ-type EFOs, one for each ZnCp* ligand, are degenerate and collectively accommodate a single alpha and a single beta electron. The equal splitting of these electrons between the two EFOs ultimately leads to an OS of 0 for each of the two symmetric ZnCp* ligands. This assignment indicates that the addition proceeds via an insertion mechanism, distinct from the behavior observed for the other compounds studied here, and corresponds to an intermediate scenario between full oxidation and full reduction, entirely consistent with the analyses presented above.

NacNac		Al	
Orb. num.	EFO occ.	Orb. num	EFO occ.
1-112	**>0.995**	1-4	**>0.995**
113	**0.958**	5	**0.994**
114	**0.943**	6	**0.769**
115	**0.912**	7	0.087
116	0.227	8	0.056
117	0.006	9	0.041
118	0.005	10	0.044
Total occ	115		6
OS	-1		+1

Table S2. Sorted gross occupations of the alpha-EFOs for each fragment in the Al(NacNac) system using the TFVC atomic definition. The occupied EFOs are in bold.

	OS [E]	OS [H/ Zn]	$\lambda_{LO}^{\sigma}/\lambda_{FU}^{\sigma}$(E)	$\lambda_{LO}^{\sigma}/\lambda_{FU}^{\sigma}$(H/Zn)	R(%)
PC_{Al}^{H2}	+3	-1	0.993/0.228	0.774/0.013	100.0
PC_{Si}^{H2}	+4	-1	0.993/0.274	0.750/0.022	97.5
PC_{Al}^{Zn2}	+3	-1	0.992/0.305	0.669/0.021	86.4
PC_{Si}^{Zn2}	+2	0	0.571/0.324	0.447/0.018	62.3

Table S3. Oxidation States, Frontier EFOs, and Reliability Index for the PC_E^{H2} and PC_E^{Zn2}.

References

1 K. Morokuma, *J. Chem. Phys.*, 1971, **55**, 1236–1244.

2 T. Ziegler and A. Rauk, *Theor. Chim. Acta*, 1977, **46**, 1–10.

3 L. Zhao, M. von Hopffgarten, D. M. Andrada and G. Frenking, *WIREs Comput. Mol. Sci.*, 2018, **8**, e1345.

4 M. Mitoraj and A. Michalak, *J. Mol. Model.*, 2007, **13**, 347–355.

5 A. Michalak, M. Mitoraj and T. Ziegler, *J. Phys. Chem. A*, 2008, **112**, 1933–1939.

6 R. F. Nalewajski and J. Mrozek, *Int. J. Quantum Chem.*, 1994, **51**, 187–200.

7 R. F. Nalewajski, J. Mrozek and A. Michalak, *Int. J. Quantum Chem.*, 1997, **61**, 589–601.

8 T. Lu and F. Chen, *J. Phys. Chem. A*, 2013, **117**, 3100–3108.

9 L. Belpassi, I. Infante, F. Tarantelli and L. Visscher, *J. Am. Chem. Soc.*, 2008, **130**, 1048–1060.

10 G. Bistoni, S. Rampino, F. Tarantelli and L. Belpassi, *J. Chem. Phys.*, 2015, **142**, 084112.

11 G. Bistoni, L. Belpassi and F. Tarantelli, *J. Chem. Theory Comput.*, 2016, **12**, 1236–1244.

12 I. Mayer, *J. Phys. Chem.*, 1996, **100**, 6249–6257.

13 E. Ramos-Cordoba, V. Postils and P. Salvador, *J. Chem. Theory Comput.*, 2015, **11**, 1501–1508.

14 W. Yang, A. J. P. White and M. R. Crimmin, *Nat. Synth.*, 2025, **4**, 995–1000.

xyz geometries

H_2

2

H	0.000000	0.000000	-0.375595
H	0.000000	0.000000	0.375595

Cp*ZnZnCp*

52

C	3.140858	-1.217391	-0.328715
C	2.998293	-0.793798	1.034959
C	2.965654	0.637859	1.055592
C	3.088129	1.107097	-0.292869
C	3.195754	-0.039327	-1.154664
ZN	1.178021	-0.123630	-0.211398
C	2.945328	-1.691360	2.232742
C	2.863158	1.496601	2.278400
C	3.177590	2.538746	-0.723379
C	3.454477	-0.010148	-2.630018
C	3.312225	-2.628922	-0.799068
ZN	-1.097191	-0.078894	-0.067823
C	-3.220647	-0.547881	-0.588342
C	-3.094206	0.881376	-0.471737
C	-2.763291	1.184887	0.891214
C	-2.685303	-0.047987	1.615989
C	-2.966745	-1.119384	0.706535
C	-3.371782	1.880289	-1.552783
C	-2.586581	2.556336	1.466280
C	-2.397939	-0.189255	3.079083
C	-3.068906	-2.571709	1.058310
C	-3.674325	-1.295712	-1.804296
H	2.372854	2.454798	2.060564
H	2.288154	1.002872	3.073367
H	3.857780	1.727863	2.693791
H	2.799072	2.680519	-1.744328
H	2.602122	3.199043	-0.060825
H	4.220490	2.896275	-0.711223
H	2.996216	0.866562	-3.106660
H	4.535221	0.031893	-2.843914
H	3.060027	-0.904488	-3.130166
H	2.781692	-3.339737	-0.151581
H	2.936139	-2.765905	-1.821581
H	4.374767	-2.923164	-0.801760
H	2.503441	-2.666775	1.989488
H	3.952707	-1.883054	2.637486
H	2.349985	-1.250409	3.043589
H	-1.877192	2.556450	2.304761
H	-3.539590	2.958277	1.847847

H	-2.213846	3.265915	0.715735
H	-1.915157	-1.149043	3.306480
H	-3.322151	-0.140988	3.678187
H	-1.736409	0.609124	3.441251
H	-4.095152	-2.839206	1.359721
H	-2.407692	-2.834543	1.894679
H	-2.803186	-3.215477	0.209380
H	-3.379146	-0.785128	-2.730424
H	-4.772319	-1.394685	-1.823639
H	-3.258039	-2.311289	-1.836665
H	-2.780057	2.796036	-1.421828
H	-4.433330	2.178138	-1.557803
H	-3.144315	1.476743	-2.548302

1Si

72

SI	0.016638	-0.050790	0.630961
N	1.333528	0.023427	-0.524799
C	2.420502	0.151403	-2.696556
H	2.333536	0.194770	-3.779843
H	3.413301	0.162554	-2.256266
N	-1.329454	-0.036833	-0.514669
C	1.292797	0.084534	-1.940757
C	-0.025025	0.066343	-2.545408
H	-0.038480	0.099660	-3.633731
C	-1.226534	0.009844	-1.915272
C	-2.505842	-0.012674	-2.698711
H	-2.292313	0.024666	-3.771831
H	-3.151610	0.837657	-2.436518
H	-3.085689	-0.922249	-2.484680
C	2.638233	0.050818	0.096057
C	3.299946	-1.166833	0.354048
C	4.530807	-1.120523	1.017992
H	5.054464	-2.049791	1.244862
C	5.096984	0.094966	1.394915
H	6.055919	0.111538	1.914486
C	4.441961	1.288439	1.105106
H	4.895909	2.236209	1.398018
C	3.206214	1.291700	0.447160
C	2.509228	2.606281	0.146650
H	1.613781	2.378849	-0.447034
C	3.391589	3.535825	-0.697549
H	4.301659	3.830127	-0.155647
H	2.842080	4.453150	-0.951455
H	3.691842	3.044170	-1.632226
C	2.052337	3.292638	1.442551
H	1.389695	2.637102	2.024409
H	1.508191	4.220475	1.216628
H	2.913194	3.548580	2.076729
C	2.688749	-2.498510	-0.043368
H	1.872850	-2.287113	-0.747704
C	3.689887	-3.407939	-0.765296

H	4.123950	-2.900443	-1.636903
H	3.185515	-4.319996	-1.113462
H	4.511465	-3.717080	-0.104062
C	2.083449	-3.201365	1.181874
H	2.862976	-3.430770	1.922875
H	1.598166	-4.143386	0.889443
H	1.332939	-2.565661	1.673383
C	-2.638939	-0.068644	0.094473
C	-3.217948	-1.311437	0.422147
C	-4.456904	-1.311231	1.074444
H	-4.920950	-2.260187	1.345870
C	-5.104151	-0.119057	1.384088
H	-6.067168	-0.138526	1.895696
C	-4.524604	1.099579	1.038451
H	-5.042080	2.027495	1.282983
C	-3.287649	1.149660	0.387406
C	-2.647848	2.484970	0.046658
H	-1.868366	2.297989	-0.705325
C	-1.958205	3.076306	1.286358
H	-2.693623	3.276583	2.078899
H	-1.451501	4.018978	1.036682
H	-1.209868	2.381108	1.694228
C	-3.639542	3.485304	-0.558283
H	-4.154638	3.062291	-1.431560
H	-3.108017	4.391375	-0.879618
H	-4.403944	3.793030	0.168636
C	-2.523023	-2.625469	0.109348
H	-1.667007	-2.402305	-0.542274
C	-1.975189	-3.266175	1.394011
H	-2.793307	-3.507917	2.087609
H	-1.285788	-2.585128	1.912400
H	-1.434286	-4.194572	1.163211
C	-3.435301	-3.599775	-0.647257
H	-4.291538	-3.912241	-0.033429
H	-2.875753	-4.504495	-0.922095
H	-3.827905	-3.147302	-1.567963

1Al

73

AL	-0.002296	0.163586	-0.984661
C	-1.273231	0.399178	1.756304
N	1.389518	0.236756	0.443446
N	-1.408012	0.190605	0.437754
C	1.236605	0.425641	1.764528
C	2.717653	0.013028	-0.066334
C	3.188174	-1.313825	-0.178061
C	-3.584939	1.039679	-0.364014
C	2.981940	2.534340	-0.421287
H	2.080037	2.525420	0.206519
C	-2.492823	0.479275	2.638120
H	-3.030115	1.421871	2.460776
H	-2.211914	0.436467	3.695281

H	-3.199927	-0.329439	2.414971
C	-2.125918	-2.526746	-0.173551
H	-1.109891	-2.131507	-0.355030
C	-2.670716	3.072624	-1.499817
H	-1.863900	2.467224	-1.939365
H	-2.298614	4.097533	-1.359261
H	-3.501465	3.101231	-2.219506
C	3.487687	1.104790	-0.520175
C	-0.020369	0.547335	2.370090
H	-0.025547	0.716225	3.444193
C	2.453349	0.490333	2.649040
H	3.016425	-0.452258	2.598309
H	2.173305	0.681422	3.689679
H	3.139055	1.278512	2.309628
C	-3.136702	2.477306	-0.160316
H	-2.264813	2.467807	0.507678
C	2.572356	3.060928	-1.805907
H	3.436024	3.079702	-2.486231
H	2.173553	4.082423	-1.727887
H	1.799984	2.419996	-2.255738
C	-5.244328	-0.542816	-1.169780
H	-6.234449	-0.738848	-1.583106
C	4.735818	0.841441	-1.095601
H	5.343711	1.671580	-1.457653
C	-2.724241	-0.044673	-0.092662
C	-3.090630	-1.374187	-0.407531
C	-2.322831	-3.691685	-1.145409
H	-3.264912	-4.226408	-0.959061
H	-1.506507	-4.416572	-1.024409
H	-2.322747	-3.346746	-2.187880
C	4.442361	-1.526440	-0.760096
H	4.820612	-2.544618	-0.860799
C	4.000843	3.468902	0.244523
H	4.305160	3.095269	1.232077
H	3.566666	4.469657	0.376040
H	4.907656	3.578369	-0.366493
C	2.356776	-2.497327	0.290782
H	1.499585	-2.101477	0.852878
C	-4.848658	0.763485	-0.897747
H	-5.531634	1.585905	-1.112346
C	-4.209966	3.357999	0.489569
H	-5.076709	3.496536	-0.171360
H	-3.799313	4.353856	0.705212
H	-4.572638	2.924732	1.432005
C	3.136708	-3.422264	1.234485
H	3.979466	-3.905689	0.720835
H	2.479497	-4.216792	1.614312
H	3.539193	-2.870574	2.095147
C	5.212460	-0.460912	-1.218625
H	6.186043	-0.646172	-1.674011
C	-4.365780	-1.598341	-0.937487
H	-4.674923	-2.613722	-1.183095
C	-2.164329	-3.019955	1.281113
H	-1.901566	-2.224020	1.987842

H	-1.452177	-3.845372	1.422355
H	-3.169119	-3.387500	1.535579
C	1.798020	-3.277417	-0.909446
H	1.198967	-2.622282	-1.559791
H	1.160158	-4.105854	-0.569299
H	2.613192	-3.697778	-1.515723

RC_{Si}^{Zn2}

124

C	3.530535	-0.438143	-3.064191
C	2.860853	0.825010	-2.981807
C	1.453584	0.574827	-2.817195
C	1.261254	-0.854772	-2.799424
C	2.548309	-1.472197	-2.948572
ZN	2.456311	-0.224187	-1.002228
ZN	3.206221	-0.373962	1.145967
C	4.040204	0.681164	2.979821
C	5.146329	0.057811	2.314138
C	4.929636	-1.357748	2.319538
C	3.689335	-1.616845	2.990369
C	3.136115	-0.354806	3.401564
C	6.342147	0.759955	1.748599
C	5.857811	-2.392693	1.762122
C	3.126924	-2.968357	3.305899
C	1.924128	-0.162459	4.260252
C	3.896479	2.142622	3.275048
C	3.497055	2.174486	-3.115767
C	0.371056	1.611051	-2.824009
C	-0.056099	-1.566720	-2.779432
C	2.804825	-2.946103	-3.020516
C	5.002722	-0.643279	-3.248305
SI	-2.518532	0.334412	-0.298872
N	-3.204493	1.589663	0.708804
C	-2.806075	2.917283	0.298475
C	-1.625238	3.477898	0.823132
C	-1.216855	4.725542	0.337727
C	-1.966442	5.401082	-0.621550
C	-3.145194	4.841839	-1.108975
C	-3.590625	3.595205	-0.656343
C	-0.785277	2.732144	1.843255
C	0.426522	2.084241	1.158221
C	-4.861189	2.978911	-1.214882
C	-6.043988	3.953855	-1.190426
N	-3.317507	-1.060264	0.435333
C	-3.012639	-2.315198	-0.206765
C	-3.617996	-2.618863	-1.446867
C	-3.297491	-3.840747	-2.050313
C	-2.402813	-4.725371	-1.454317
C	-1.784307	-4.390046	-0.252564
C	-2.064889	-3.177124	0.383712
C	-4.583868	-1.651956	-2.113928

C	-6.007004	-1.791814	-1.553415
C	-1.292494	-2.745819	1.617576
C	-0.019518	-2.008968	1.172997
C	-4.202846	-1.027708	1.524223
C	-4.771065	-2.334167	1.998855
C	-4.539436	0.138076	2.137586
C	-4.095558	1.478350	1.804966
C	-4.521476	2.565713	2.500548
C	-4.583358	-1.749098	-3.640771
C	-0.957057	-3.893492	2.573539
C	-0.348726	3.618670	3.015068
C	-4.617907	2.432381	-2.630971
H	-5.231851	0.072812	2.975434
H	-5.242606	-2.889183	1.175943
H	-5.516784	-2.157377	2.781062
H	-3.987765	-2.989453	2.405983
H	-4.197443	3.572595	2.253185
H	-5.211778	2.432380	3.330468
H	-3.749381	-4.102590	-3.006340
H	-2.171791	-5.674205	-1.940131
H	-1.061044	-5.075801	0.188582
H	-4.241072	-0.631511	-1.865927
H	-5.170561	-0.922808	-4.063769
H	-3.564226	-1.688993	-4.045611
H	-5.037994	-2.686135	-3.992436
H	-6.685044	-1.087557	-2.055881
H	-6.386859	-2.810639	-1.718078
H	-6.039258	-1.581357	-0.477719
H	-1.909110	-2.022601	2.169182
H	-1.855764	-4.456803	2.860359
H	-0.497997	-3.496117	3.489119
H	-0.240536	-4.599035	2.130376
H	0.654668	-2.684823	0.628171
H	0.527778	-1.603503	2.034542
H	-0.251876	-1.169889	0.500916
H	-0.296203	5.173397	0.713133
H	-1.630502	6.370871	-0.991429
H	-3.725875	5.380422	-1.858718
H	-1.410039	1.927522	2.254992
H	0.174615	3.014111	3.769243
H	-1.213989	4.091862	3.498052
H	0.340945	4.411405	2.692681
H	0.117412	1.427002	0.332637
H	1.005827	1.476973	1.866920
H	1.097379	2.847188	0.737231
H	-5.125691	2.130868	-0.569237
H	-3.786512	1.711920	-2.640453
H	-5.517550	1.928124	-3.012074
H	-4.359206	3.245401	-3.324530
H	-6.214794	4.336495	-0.175499
H	-5.880013	4.811788	-1.857594
H	-6.958768	3.444702	-1.524493
H	6.753405	0.225015	0.881800
H	7.151275	0.841899	2.492824

H	6.097361	1.779711	1.422957
H	5.313938	-3.285997	1.426720
H	6.592473	-2.723934	2.514382
H	6.425279	-2.008111	0.903901
H	2.033998	-2.943517	3.403493
H	3.528685	-3.357506	4.255788
H	3.370714	-3.702720	2.526279
H	1.417648	0.789353	4.050899
H	2.191734	-0.155463	5.329579
H	1.188360	-0.965744	4.119853
H	4.376857	2.761331	2.505352
H	4.361457	2.404627	4.239682
H	2.842634	2.446294	3.331090
H	2.936181	2.942145	-2.566267
H	3.539730	2.496515	-4.169412
H	4.527359	2.178460	-2.734974
H	-0.525791	1.265999	-2.292717
H	0.067850	1.860728	-3.854337
H	0.694712	2.545780	-2.346387
H	-0.002608	-2.528716	-2.252178
H	-0.408366	-1.778593	-3.802902
H	-0.832511	-0.967977	-2.285856
H	3.794423	-3.206789	-2.621079
H	2.768113	-3.312689	-4.059750
H	2.055658	-3.513604	-2.452113
H	5.582799	0.191203	-2.831751
H	5.271345	-0.724825	-4.314419
H	5.347176	-1.563946	-2.757613

TS'$_{Si}^{Zn2}$

124

C	-2.895588	0.093829	3.470834
C	-2.064922	1.241184	3.296047
C	-0.727409	0.791779	3.028809
C	-0.744235	-0.658052	3.038766
C	-2.091558	-1.073727	3.308485
ZN	-1.829844	0.066750	1.274602
ZN	-3.108984	0.084435	-0.615687
C	-4.591146	1.272377	-1.853890
C	-5.377091	0.758519	-0.774470
C	-5.346459	-0.668496	-0.836844
C	-4.543852	-1.052451	-1.957717
C	-4.070682	0.151021	-2.596534
C	-6.105410	1.577319	0.246265
C	-6.036083	-1.605687	0.105947
C	-4.332654	-2.449563	-2.454177
C	-3.367555	0.226222	-3.916341
C	-4.424408	2.715011	-2.220650
C	-2.499755	2.670066	3.406623
C	0.495677	1.650091	2.930580
C	0.456766	-1.548244	2.969561
C	-2.558893	-2.490613	3.437546

C	-4.363338	0.107258	3.766138
SI	2.217324	-0.085735	-0.171499
N	3.226484	1.124380	-0.936617
C	2.908964	2.470537	-0.514762
C	1.870252	3.167339	-1.165379
C	1.522169	4.435225	-0.685161
C	2.196286	5.005744	0.390369
C	3.239115	4.315001	1.001309
C	3.618144	3.042093	0.561820
C	1.117347	2.574676	-2.342624
C	-0.333909	2.259273	-1.956491
C	4.763229	2.319212	1.247810
C	6.077895	3.101193	1.114509
N	2.940518	-1.530648	-0.902230
C	2.364364	-2.770023	-0.435035
C	2.848440	-3.386751	0.739848
C	2.169190	-4.526147	1.199090
C	1.066403	-5.044163	0.530016
C	0.619607	-4.434868	-0.638596
C	1.257396	-3.296913	-1.139809
C	4.090042	-2.991953	1.540942
C	4.379046	-1.505271	1.766238
C	0.729158	-2.618638	-2.392919
C	-0.466276	-1.724372	-2.041916
C	3.939046	-1.542184	-1.885010
C	4.291194	-2.865100	-2.502577
C	4.541949	-0.398862	-2.307904
C	4.262085	0.962038	-1.890324
C	4.941252	2.019955	-2.406750
C	5.345874	-3.684670	0.982229
C	0.365078	-3.610238	-3.503827
C	1.180908	3.485280	-3.575995
C	4.439981	2.031721	2.720804
H	5.307185	-0.500119	-3.076096
H	4.530703	-3.622368	-1.745482
H	5.146407	-2.752223	-3.177105
H	3.443866	-3.262061	-3.081380
H	4.722642	3.043720	-2.117421
H	5.727587	1.845015	-3.137593
H	2.519696	-5.008991	2.113425
H	0.553705	-5.924264	0.920200
H	-0.243814	-4.844190	-1.163425
H	3.912423	-3.423049	2.539009
H	6.185004	-3.562769	1.681577
H	5.177621	-4.758618	0.827856
H	5.645400	-3.238574	0.024482
H	5.141956	-1.401688	2.551014
H	4.768881	-1.017448	0.864151
H	3.487126	-0.959501	2.104901
H	1.521601	-1.964220	-2.780448
H	1.205060	-4.277724	-3.740397
H	0.092825	-3.064442	-4.417617
H	-0.495010	-4.235847	-3.227144
H	-1.297056	-2.321610	-1.640461

H	-0.824006	-1.181159	-2.927472
H	-0.201733	-0.983842	-1.273156
H	0.708659	4.983523	-1.162349
H	1.909778	5.994039	0.752738
H	3.767964	4.770202	1.839979
H	1.608493	1.628780	-2.608771
H	0.681027	3.003461	-4.428222
H	2.221819	3.691033	-3.858297
H	0.678398	4.445813	-3.393301
H	-0.380911	1.587589	-1.088264
H	-0.860638	1.772877	-2.789566
H	-0.882601	3.175651	-1.694977
H	4.900627	1.356856	0.738416
H	3.514029	1.450907	2.819884
H	5.255901	1.462969	3.188250
H	4.309974	2.965262	3.286637
H	6.320301	3.278556	0.058750
H	6.017494	4.073627	1.624064
H	6.903864	2.534748	1.567536
H	-6.180849	1.050563	1.206855
H	-7.133003	1.810019	-0.077594
H	-5.600578	2.534959	0.432697
H	-5.497120	-2.558474	0.195601
H	-7.058875	-1.841738	-0.230652
H	-6.119537	-1.176431	1.113577
H	-3.395549	-2.543552	-3.018427
H	-5.148803	-2.763868	-3.125368
H	-4.297499	-3.173879	-1.628830
H	-2.734445	1.119998	-3.993969
H	-4.091553	0.270391	-4.746770
H	-2.730237	-0.650350	-4.091640
H	-4.463009	3.365219	-1.336311
H	-5.221623	3.050056	-2.904546
H	-3.466252	2.898744	-2.724413
H	-1.857624	3.333773	2.812490
H	-2.459108	3.026962	4.448996
H	-3.532507	2.808730	3.057981
H	1.242897	1.203395	2.260795
H	0.970397	1.787941	3.916213
H	0.267255	2.648659	2.534639
H	0.219655	-2.530917	2.541224
H	0.886852	-1.719022	3.970737
H	1.241140	-1.106187	2.341742
H	-3.590875	-2.611867	3.079833
H	-2.538317	-2.830783	4.486218
H	-1.924580	-3.177672	2.861627
H	-4.841912	1.024242	3.397295
H	-4.560501	0.052780	4.849424
H	-4.877596	-0.746534	3.303376

INT_{Si}^{Zn2}

124

SI	-1.345522	-0.149160	-0.059237
ZN	2.258550	0.427869	0.448557
ZN	0.863560	-0.034996	-1.402127
N	-1.263240	-1.453206	1.160622
C	-1.318258	-1.214147	2.541614
C	-1.469570	0.034616	3.064936
H	-1.477141	0.106271	4.151470
C	-1.736500	1.281463	2.374353
N	-1.744590	1.236179	0.960480
C	-1.214972	-2.392814	3.464709
H	-1.908247	-3.192882	3.170771
H	-1.444092	-2.085902	4.490389
H	-0.206755	-2.824814	3.452200
C	-2.018249	2.416607	3.067317
H	-2.261819	3.351677	2.571965
H	-2.020794	2.391928	4.154424
C	-1.336278	-2.809544	0.650051
C	-2.572634	-3.268478	0.119218
C	-2.646654	-4.571298	-0.382766
H	-3.587170	-4.934240	-0.792941
C	-1.543468	-5.418318	-0.367183
H	-1.623500	-6.432254	-0.761040
C	-0.339924	-4.957227	0.143152
H	0.529065	-5.615529	0.138374
C	-0.205791	-3.657637	0.650269
C	-3.822856	-2.402732	0.117683
H	-3.504639	-1.370843	-0.119603
C	-4.838222	-2.802860	-0.955366
H	-5.645274	-2.062305	-0.998080
H	-4.373118	-2.860156	-1.948650
H	-5.302310	-3.774693	-0.735782
C	-4.481510	-2.374819	1.505522
H	-5.386776	-1.751914	1.487716
H	-4.772270	-3.390814	1.809694
H	-3.806765	-1.966192	2.267033
C	1.173322	-3.240358	1.130601
H	1.103419	-2.227577	1.553740
C	1.724117	-4.198326	2.199444
H	1.049288	-4.306037	3.057890
H	2.694112	-3.841382	2.570404
H	1.887853	-5.200771	1.780835
C	2.145882	-3.188757	-0.054391
H	2.246569	-4.179253	-0.519799
H	3.137528	-2.861057	0.274156
H	1.795572	-2.485557	-0.817854
C	-2.302373	2.374654	0.257787
C	-1.484818	3.452766	-0.137646
C	-2.058606	4.487489	-0.888204
H	-1.430777	5.316571	-1.216584
C	-3.409453	4.485040	-1.210656
H	-3.836235	5.299831	-1.797080
C	-4.217935	3.444589	-0.764422
H	-5.284228	3.459825	-0.991327
C	-3.690091	2.381408	-0.024870

C	-0.023562	3.572731	0.250671
H	0.249593	2.680331	0.832701
C	0.184865	4.812124	1.135830
H	1.243009	4.921422	1.408494
H	-0.401382	4.741614	2.060727
H	-0.112881	5.728310	0.606835
C	0.878363	3.630238	-0.985843
H	0.760893	2.725388	-1.592730
H	1.932055	3.702534	-0.694165
H	0.638552	4.498189	-1.616640
C	-4.634128	1.315247	0.499421
H	-4.025890	0.494258	0.900605
C	-5.535208	0.747745	-0.602265
H	-4.942421	0.325792	-1.425058
H	-6.178621	-0.043593	-0.194964
H	-6.194951	1.519335	-1.021918
C	-5.472757	1.867072	1.664328
H	-4.822533	2.243378	2.464239
H	-6.119537	2.689036	1.324199
H	-6.114799	1.077289	2.080103
C	4.548377	0.866545	0.860977
C	4.181974	-0.290377	1.617207
C	3.135990	0.085247	2.523777
C	2.878086	1.489328	2.344914
C	3.764036	1.971254	1.323027
C	5.643178	0.935725	-0.158612
H	5.775048	-0.022967	-0.678250
H	6.612194	1.187682	0.304192
H	5.443010	1.702377	-0.919750
C	4.916605	-1.593437	1.586059
H	4.349186	-2.398746	2.067456
H	5.874712	-1.509083	2.125045
H	5.156126	-1.915315	0.562709
C	2.526409	-0.749428	3.607927
H	1.445112	-0.572292	3.696706
H	2.975084	-0.513275	4.587077
H	2.676288	-1.820927	3.433571
C	1.971305	2.298167	3.220748
H	1.778869	3.291736	2.802322
H	2.418550	2.443924	4.218127
H	0.995896	1.814736	3.368306
C	3.986241	3.397293	0.928674
H	4.152690	3.510891	-0.151808
H	4.877963	3.804472	1.433199
H	3.140524	4.036161	1.206301
C	1.728637	0.600986	-3.449232
C	0.299346	0.698515	-3.517293
C	-0.236810	-0.627863	-3.393189
C	0.855821	-1.539108	-3.223739
C	2.070294	-0.781851	-3.265187
C	2.715326	1.715077	-3.611656
H	2.244592	2.696686	-3.478185
H	3.172995	1.702446	-4.613972
H	3.534044	1.641006	-2.881120

C	-0.526528	1.912077	-3.820668
H	-1.344499	2.049280	-3.098112
H	-0.986129	1.829224	-4.818733
H	0.075567	2.828116	-3.814390
C	-1.678986	-0.979706	-3.451146
H	-1.828560	-2.055780	-3.605020
H	-2.198982	-0.436356	-4.252870
H	-2.187575	-0.710514	-2.496829
C	0.723753	-3.032126	-3.200373
H	1.677681	-3.518052	-2.965274
H	0.402233	-3.407123	-4.185406
H	-0.013021	-3.378635	-2.462396
C	3.464161	-1.321460	-3.182736
H	4.134137	-0.620882	-2.665079
H	3.891216	-1.500074	-4.183198
H	3.499166	-2.273887	-2.637824

TS_{Si}^{Zn2}

124

SI	-0.636260	-0.022426	0.216283
ZN	1.940984	0.533022	0.525577
ZN	0.644411	-0.277177	-1.632700
N	-0.738351	-1.271743	1.470489
C	-0.676766	-0.861983	2.806796
C	-0.735236	0.452985	3.171536
H	-0.586960	0.663939	4.229249
C	-1.055475	1.618290	2.364673
N	-1.334479	1.392453	0.993464
C	-0.500706	-1.924109	3.853042
H	-1.238127	-2.729836	3.733609
H	-0.611895	-1.491295	4.852484
H	0.490759	-2.392227	3.784219
C	-1.151280	2.855501	2.918343
H	-1.425085	3.726139	2.328070
H	-0.996350	2.983330	3.987231
C	-0.920240	-2.659492	1.109868
C	-2.219693	-3.119773	0.776044
C	-2.391029	-4.462596	0.426492
H	-3.384367	-4.826505	0.169493
C	-1.317911	-5.347124	0.404234
H	-1.471942	-6.391090	0.128723
C	-0.050037	-4.886095	0.724431
H	0.793124	-5.576135	0.689624
C	0.176357	-3.548545	1.075873
C	-3.432874	-2.212443	0.853774
H	-3.108882	-1.199315	0.565231
C	-4.559536	-2.626524	-0.097763
H	-5.346598	-1.863956	-0.099977
H	-4.193974	-2.747608	-1.126165
H	-5.028337	-3.570961	0.212177
C	-3.944492	-2.149096	2.303963
H	-4.851156	-1.534169	2.370110

H	-4.191167	-3.159185	2.661412
H	-3.194902	-1.717190	2.977469
C	1.607698	-3.129799	1.357259
H	1.599841	-2.096019	1.736122
C	2.273056	-4.039737	2.400986
H	1.704361	-4.067836	3.339908
H	3.288712	-3.691621	2.623779
H	2.354897	-5.071743	2.033931
C	2.408703	-3.136796	0.049591
H	2.420625	-4.141611	-0.396100
H	3.446235	-2.825258	0.219562
H	1.963477	-2.448256	-0.680651
C	-2.126930	2.360581	0.273924
C	-1.518777	3.324396	-0.557754
C	-2.333715	4.176836	-1.315906
H	-1.867697	4.901663	-1.984350
C	-3.716099	4.134612	-1.212971
H	-4.335325	4.809707	-1.805252
C	-4.304767	3.235146	-0.329389
H	-5.389521	3.224906	-0.225725
C	-3.538505	2.336736	0.418823
C	-0.018314	3.532064	-0.634603
H	0.461153	2.842229	0.077740
C	0.342370	4.969502	-0.230134
H	1.426207	5.128611	-0.302170
H	0.031856	5.185320	0.800045
H	-0.141223	5.699076	-0.893979
C	0.516987	3.224711	-2.034723
H	0.269520	2.197037	-2.330822
H	1.606891	3.339260	-2.071283
H	0.076655	3.897703	-2.784434
C	-4.248832	1.403809	1.381906
H	-3.551485	0.594611	1.635810
C	-5.508912	0.791068	0.759569
H	-5.295024	0.330172	-0.213194
H	-5.931889	0.026162	1.423691
H	-6.290253	1.548467	0.608428
C	-4.611152	2.129347	2.689613
H	-3.716939	2.548202	3.166442
H	-5.319373	2.947358	2.490603
H	-5.086629	1.429516	3.392046
C	4.256371	0.675410	0.430042
C	3.919823	0.120264	1.705535
C	3.164705	1.097981	2.427848
C	3.079936	2.279680	1.622440
C	3.746819	2.018573	0.384729
C	5.114195	0.030955	-0.614764
H	4.988347	-1.059897	-0.631336
H	6.184224	0.232501	-0.439691
H	4.878029	0.406672	-1.619646
C	4.460970	-1.140443	2.296422
H	3.756476	-1.597834	3.002182
H	5.386170	-0.932148	2.859642
H	4.715256	-1.886784	1.534513

C	2.694107	0.979019	3.842986
H	1.826670	1.624433	4.026756
H	3.486437	1.272595	4.552428
H	2.397821	-0.048935	4.090408
C	2.505447	3.577521	2.098363
H	2.587855	4.358674	1.335947
H	3.047237	3.933164	2.989337
H	1.445025	3.488431	2.377146
C	4.015172	2.996382	-0.717024
H	3.900426	2.541904	-1.711127
H	5.044933	3.385555	-0.659433
H	3.339690	3.858704	-0.668424
C	0.935052	-0.069532	-3.755720
C	-0.480118	-0.045262	-3.981260
C	-1.007122	-1.322044	-3.665976
C	0.045941	-2.173226	-3.250964
C	1.274717	-1.429891	-3.297887
C	1.946202	0.901627	-4.291817
H	1.555102	1.925619	-4.322642
H	2.239248	0.628239	-5.319064
H	2.861765	0.918160	-3.685635
C	-1.290219	1.136046	-4.418144
H	-1.913076	1.537533	-3.602677
H	-1.972957	0.860041	-5.235415
H	-0.654918	1.953993	-4.779385
C	-2.462469	-1.655940	-3.596889
H	-2.634490	-2.737416	-3.678278
H	-3.033236	-1.159313	-4.393996
H	-2.897183	-1.326215	-2.636597
C	-0.108914	-3.600259	-2.825722
H	0.839177	-4.021681	-2.471864
H	-0.456178	-4.226787	-3.662665
H	-0.839679	-3.711989	-2.012356
C	2.660969	-2.002783	-3.363273
H	3.416166	-1.311078	-2.968099
H	2.933091	-2.225625	-4.408249
H	2.743489	-2.938246	-2.797187

PC_{Si}^{Zn2}

124

SI	0.198117	0.009878	0.163460
ZN	2.511667	0.007460	-0.311058
ZN	-1.514945	0.001698	-1.375667
N	0.005580	-1.365848	1.297647
C	0.812616	-1.218356	2.426899
C	1.230497	0.013281	2.859459
H	1.938556	0.018186	3.685726
C	0.801955	1.339170	2.420184
N	-0.018181	1.385331	1.277149
C	1.300446	-2.472154	3.095878
H	0.464132	-3.115827	3.400999

H	1.897282	-2.230049	3.981710
H	1.915781	-3.065802	2.402673
C	1.176307	2.458095	3.099233
H	0.870396	3.448941	2.771373
H	1.759811	2.371339	4.012868
C	-0.689434	-2.593178	1.028206
C	-1.949985	-2.835185	1.638242
C	-2.597171	-4.046289	1.368332
H	-3.560973	-4.252576	1.830076
C	-2.052447	-4.988091	0.500537
H	-2.585115	-5.917790	0.296008
C	-0.833924	-4.729878	-0.110326
H	-0.416019	-5.463170	-0.799940
C	-0.127224	-3.548582	0.148918
C	-2.629237	-1.806319	2.532651
H	-2.454737	-0.827551	2.058642
C	-4.142262	-2.026266	2.653185
H	-4.599193	-1.175084	3.171333
H	-4.629931	-2.133219	1.677553
H	-4.367707	-2.923057	3.248127
C	-2.038481	-1.741322	3.951960
H	-2.619317	-1.034238	4.561484
H	-2.093208	-2.727277	4.436067
H	-0.998536	-1.401713	3.956443
C	1.209269	-3.339960	-0.542084
H	1.734497	-2.526640	-0.016818
C	2.096273	-4.589731	-0.483668
H	2.283416	-4.904718	0.551606
H	3.063505	-4.389165	-0.961770
H	1.642835	-5.434859	-1.018805
C	1.013256	-2.898133	-1.999835
H	0.510043	-3.687273	-2.576349
H	1.980966	-2.686563	-2.473993
H	0.396593	-1.990721	-2.064459
C	-0.762220	2.564686	0.957189
C	-0.329356	3.433132	-0.071885
C	-1.154381	4.502631	-0.446493
H	-0.841119	5.157997	-1.260328
C	-2.346948	4.761438	0.217233
H	-2.972855	5.603182	-0.082532
C	-2.726994	3.946525	1.279876
H	-3.650951	4.165598	1.816574
C	-1.960695	2.841553	1.661460
C	1.021296	3.286546	-0.751284
H	1.586387	2.518795	-0.200200
C	1.819020	4.596736	-0.673978
H	2.801271	4.473984	-1.149189
H	1.976908	4.905243	0.367542
H	1.301853	5.412109	-1.198113
C	0.891465	2.832123	-2.210576
H	0.344657	1.881992	-2.288578
H	1.881719	2.693059	-2.662809
H	0.347235	3.580656	-2.802798
C	-2.423991	1.993243	2.830255

H	-1.789849	1.097269	2.858128
C	-3.881759	1.546905	2.672867
H	-4.027510	0.967703	1.751482
H	-4.178628	0.920770	3.524679
H	-4.566951	2.405196	2.643707
C	-2.234991	2.741822	4.159425
H	-1.186171	3.034460	4.293606
H	-2.859355	3.647250	4.189859
H	-2.525506	2.099981	5.003749
C	4.466738	-0.617802	-1.172359
C	4.537579	-1.053900	0.194778
C	4.507270	0.102440	1.029960
C	4.434942	1.259393	0.199512
C	4.405899	0.825376	-1.168188
C	4.632406	-1.470028	-2.393487
H	4.325787	-2.508248	-2.211583
H	5.684964	-1.496325	-2.721194
H	4.041439	-1.093995	-3.239958
C	4.754537	-2.452423	0.679591
H	4.099985	-2.707117	1.525104
H	5.791558	-2.589475	1.026674
H	4.579094	-3.190581	-0.110142
C	4.634063	0.114932	2.519622
H	4.084194	0.956523	2.960507
H	5.689050	0.212367	2.827016
H	4.248437	-0.810048	2.967194
C	4.475472	2.667670	0.707236
H	4.375242	3.394998	-0.105433
H	5.432650	2.872460	1.212558
H	3.674268	2.864871	1.435456
C	4.505279	1.690966	-2.386736
H	3.927657	1.286452	-3.229303
H	5.551051	1.778162	-2.725264
H	4.142401	2.708439	-2.194475
C	-2.021860	-0.253851	-3.579937
C	-2.543733	1.004281	-3.122682
C	-3.503196	0.739802	-2.086813
C	-3.578682	-0.683938	-1.910154
C	-2.663813	-1.296900	-2.831747
C	-1.033250	-0.444788	-4.688900
H	-0.349792	0.410464	-4.773040
H	-1.537682	-0.555602	-5.662295
H	-0.420585	-1.343334	-4.536503
C	-2.267447	2.353434	-3.707152
H	-2.224844	3.130968	-2.932651
H	-3.061031	2.639506	-4.416654
H	-1.317495	2.374367	-4.254727
C	-4.348939	1.772364	-1.409935
H	-4.749931	1.407522	-0.456303
H	-5.208528	2.053342	-2.040351
H	-3.781969	2.687360	-1.196276
C	-4.523270	-1.420631	-1.015980
H	-4.061659	-2.325016	-0.598319
H	-5.424917	-1.735907	-1.566327

H	-4.856701	-0.794988	-0.178674
C	-2.506485	-2.772216	-3.030493
H	-1.596208	-3.008236	-3.594308
H	-3.357905	-3.184114	-3.596394
H	-2.454721	-3.313394	-2.075945

INT_{Al}^{Zn2}

125

AL	-1.026971	-0.044192	-0.100641
ZN	2.613847	0.206500	0.474164
ZN	1.065849	-0.084511	-1.348595
N	-1.407143	-1.408980	1.206611
C	-1.493978	-1.166554	2.524875
C	-1.469005	0.133715	3.059886
H	-1.488684	0.200448	4.145020
C	-1.583760	1.349802	2.369084
N	-1.565135	1.420966	1.027325
C	-1.638491	-2.312002	3.487502
H	-2.362145	-3.049174	3.117886
H	-1.948118	-1.956783	4.475042
H	-0.679129	-2.837199	3.593491
C	-1.727475	2.609207	3.177342
H	-2.472110	3.285467	2.741847
H	-0.769181	3.150035	3.175758
H	-1.994523	2.386886	4.214854
C	-1.589872	-2.749941	0.698152
C	-2.832469	-3.074477	0.098312
C	-3.020959	-4.372047	-0.386226
H	-3.970549	-4.639283	-0.846312
C	-2.013118	-5.327852	-0.303182
H	-2.181014	-6.335665	-0.684326
C	-0.784207	-4.979373	0.237934
H	0.019096	-5.716110	0.261309
C	-0.540931	-3.692227	0.734799
C	-3.951263	-2.052771	-0.020420
H	-3.473670	-1.076681	-0.229727
C	-4.903526	-2.336651	-1.184801
H	-4.355390	-2.483131	-2.124426
H	-5.516542	-3.229414	-0.998742
H	-5.591221	-1.493308	-1.318576
C	-4.731883	-1.914335	1.295811
H	-4.089037	-1.588010	2.121923
H	-5.542362	-1.180348	1.188647
H	-5.181215	-2.879015	1.571898
C	0.865517	-3.362177	1.201023
H	0.871918	-2.342671	1.613690
C	1.364983	-4.333219	2.280093
H	2.379226	-4.061076	2.600334
H	0.718119	-4.337760	3.167502
H	1.410808	-5.361834	1.897085
C	1.811958	-3.375986	-0.008745
H	2.815608	-3.041551	0.279403

H	1.888119	-4.387636	-0.432934
H	1.445994	-2.705499	-0.797179
C	-1.985807	2.624958	0.347024
C	-1.053331	3.610801	-0.035259
C	-1.511592	4.709240	-0.773689
H	-0.797750	5.472144	-1.086005
C	-2.850951	4.848826	-1.113570
H	-3.185324	5.714685	-1.686140
C	-3.762183	3.875096	-0.719290
H	-4.813902	3.988150	-0.982765
C	-3.353747	2.749295	0.002622
C	0.420000	3.548941	0.322315
H	0.598683	2.637367	0.912641
C	0.820156	4.767413	1.168708
H	0.228836	4.836393	2.091450
H	1.881349	4.716866	1.443835
H	0.671614	5.700354	0.607737
C	1.287630	3.458026	-0.938816
H	0.995156	2.595187	-1.551314
H	1.185963	4.361620	-1.556899
H	2.344694	3.339404	-0.670631
C	-4.391819	1.720459	0.418237
H	-3.856886	0.822504	0.755386
C	-5.299288	1.315550	-0.748668
H	-6.001131	0.534533	-0.427582
H	-5.899053	2.162069	-1.108793
H	-4.713528	0.931279	-1.594542
C	-5.228003	2.224829	1.605511
H	-5.957994	1.462327	1.911858
H	-4.598536	2.458987	2.473388
H	-5.779380	3.135761	1.331626
C	4.879814	0.441298	1.057675
C	4.374543	-0.685483	1.781599
C	3.355576	-0.216417	2.671133
C	3.243092	1.201936	2.507337
C	4.192443	1.610278	1.516312
C	5.996817	0.415259	0.060014
H	6.032065	-0.538737	-0.483913
H	5.893542	1.216677	-0.684408
H	6.979275	0.548173	0.543877
C	4.922934	-2.076434	1.697262
H	4.230352	-2.815662	2.118910
H	5.135649	-2.375088	0.660663
H	5.869272	-2.167021	2.255938
C	2.609742	-1.011971	3.698288
H	2.665535	-2.088570	3.500617
H	3.018307	-0.846771	4.709354
H	1.545137	-0.736206	3.735458
C	2.360887	2.082491	3.338618
H	2.200866	3.060995	2.871403
H	1.373718	1.625576	3.502126
H	2.797279	2.266536	4.334858
C	4.517808	3.013765	1.108132
H	3.706231	3.708589	1.354804

H	5.422356	3.379519	1.621761
H	4.708395	3.097304	0.028525
C	1.493477	-1.040210	-3.272866
C	1.421078	0.407081	-3.466682
C	0.051932	0.728579	-3.736156
C	-0.700095	-0.470852	-3.715911
C	0.164324	-1.553832	-3.425483
C	2.770993	-1.830115	-3.314827
H	3.571596	-1.344055	-2.739318
H	3.134871	-1.946306	-4.350141
H	2.640082	-2.838150	-2.899377
C	2.615770	1.284109	-3.716093
H	2.383301	2.344946	-3.553869
H	2.978871	1.181528	-4.753145
H	3.453145	1.031078	-3.050618
C	-0.518023	2.097889	-3.953864
H	-1.136480	2.130255	-4.864300
H	0.269994	2.853813	-4.066209
H	-1.163433	2.419754	-3.119815
C	-2.190225	-0.542515	-3.718932
H	-2.586553	-0.390868	-2.690290
H	-2.559048	-1.519753	-4.060715
H	-2.643921	0.235532	-4.349103
C	-0.264475	-2.981401	-3.265035
H	0.591053	-3.644192	-3.082925
H	-0.777608	-3.349469	-4.167736
H	-0.964340	-3.114673	-2.424792

TS_{Al}^{Zn2}

125

AL	-0.791022	0.031108	0.015949
ZN	1.936201	-0.434646	-0.888763
ZN	0.924155	0.045640	1.569807
N	-1.261554	1.463046	-1.164108
C	-1.493854	1.218478	-2.462505
C	-1.606091	-0.088387	-2.979569
H	-1.706764	-0.159254	-4.059988
C	-1.803365	-1.282229	-2.270323
N	-1.702127	-1.357854	-0.927936
C	-1.654728	2.363503	-3.422397
H	-2.273341	3.159232	-2.989367
H	-2.097575	2.026369	-4.364366
H	-0.673360	2.808724	-3.640013
C	-2.149967	-2.521175	-3.048481
H	-3.030960	-3.024742	-2.633443
H	-1.318466	-3.237591	-2.978619
H	-2.326928	-2.290463	-4.103069
C	-1.312933	2.815381	-0.652651
C	-2.468463	3.231605	0.051689
C	-2.521405	4.542858	0.533341
H	-3.405411	4.879002	1.072587
C	-1.464075	5.426961	0.343667

H	-1.525942	6.446704	0.725375
C	-0.321636	4.991977	-0.312493
H	0.522483	5.672131	-0.427725
C	-0.216637	3.687190	-0.811122
C	-3.649960	2.303609	0.265894
H	-3.246105	1.281815	0.369913
C	-4.426093	2.613960	1.549421
H	-3.755592	2.660984	2.417974
H	-4.964986	3.568890	1.479212
H	-5.173519	1.834245	1.735349
C	-4.583048	2.319655	-0.955787
H	-4.067963	1.986996	-1.865405
H	-5.446436	1.659944	-0.796579
H	-4.960044	3.337530	-1.130371
C	1.109270	3.252951	-1.402441
H	0.997119	2.243117	-1.825766
C	1.594201	4.197461	-2.510386
H	2.528980	3.826923	-2.950175
H	0.855660	4.296596	-3.317513
H	1.795841	5.204132	-2.119121
C	2.136906	3.157996	-0.265898
H	3.088516	2.757641	-0.629394
H	2.317372	4.146898	0.179938
H	1.770708	2.492925	0.527201
C	-2.266390	-2.492771	-0.231575
C	-1.457555	-3.584628	0.141520
C	-2.039644	-4.635456	0.861439
H	-1.417764	-5.477157	1.167471
C	-3.390792	-4.633879	1.181016
H	-3.827308	-5.466732	1.733502
C	-4.182530	-3.559512	0.790533
H	-5.244371	-3.561215	1.037098
C	-3.643430	-2.471101	0.097060
C	0.012914	-3.684662	-0.212432
H	0.279055	-2.826574	-0.850243
C	0.289562	-4.976314	-0.997724
H	-0.325931	-5.034911	-1.905757
H	1.343653	-5.036186	-1.294546
H	0.070780	-5.864718	-0.389741
C	0.880717	-3.604075	1.049813
H	0.672749	-2.678707	1.605826
H	0.680705	-4.451198	1.721725
H	1.945676	-3.613226	0.788588
C	-4.559542	-1.319484	-0.279389
H	-3.929641	-0.485766	-0.615828
C	-5.377160	-0.841378	0.927117
H	-5.994379	0.023234	0.650635
H	-6.058555	-1.623628	1.287051
H	-4.724339	-0.552079	1.761091
C	-5.493140	-1.690590	-1.442537
H	-6.139645	-0.839372	-1.698893
H	-4.932421	-1.969871	-2.343222
H	-6.138326	-2.537648	-1.168419
C	4.341106	-0.801841	-1.393375

C	3.948718	0.289596	-2.224970
C	2.907066	-0.164837	-3.089134
C	2.678510	-1.554679	-2.814782
C	3.575923	-1.948305	-1.770869
C	5.462621	-0.791953	-0.400710
H	5.603959	0.202037	0.045561
H	5.286870	-1.501090	0.420235
H	6.422838	-1.073651	-0.866416
C	4.657857	1.605220	-2.295289
H	4.030030	2.393541	-2.727902
H	4.995373	1.950407	-1.307735
H	5.558655	1.532629	-2.928400
C	2.233414	0.618876	-4.174430
H	2.317765	1.700454	-4.006608
H	2.672784	0.410043	-5.164581
H	1.162031	0.378477	-4.240014
C	1.750691	-2.439433	-3.590039
H	1.479816	-3.341975	-3.027495
H	0.818233	-1.915833	-3.846259
H	2.204077	-2.773370	-4.538720
C	3.826562	-3.336880	-1.273302
H	2.985246	-4.005039	-1.486243
H	4.715848	-3.777271	-1.755229
H	4.007999	-3.364066	-0.189217
C	2.247434	0.943672	3.024731
C	2.016172	-0.466195	3.349516
C	0.772951	-0.535060	4.066701
C	0.247142	0.774987	4.176835
C	1.132442	1.684333	3.540435
C	3.570839	1.493519	2.577409
H	4.008042	0.901443	1.761976
H	4.296041	1.496735	3.408420
H	3.479958	2.525378	2.216719
C	3.073337	-1.530087	3.273882
H	2.638851	-2.537996	3.262496
H	3.754741	-1.474702	4.139524
H	3.686172	-1.427890	2.367999
C	0.109881	-1.781919	4.568369
H	0.010352	-1.771015	5.665432
H	0.683388	-2.678642	4.301445
H	-0.903385	-1.902914	4.155102
C	-1.116080	1.120176	4.688806
H	-1.865198	1.096337	3.876281
H	-1.143897	2.130076	5.122064
H	-1.451599	0.413196	5.460015
C	0.902004	3.159746	3.404164
H	1.763806	3.663897	2.949097
H	0.727402	3.626276	4.386157
H	0.023488	3.386789	2.779907

PC_{Al}^{Zn2}

125

AL	0.148603	-0.010788	0.139993
ZN	2.533952	0.016225	-0.499024
ZN	-1.666307	-0.163635	-1.399664
N	0.023245	-1.385836	1.508024
C	0.781499	-1.165021	2.589272
C	1.241166	0.126007	2.932425
H	1.928063	0.170950	3.774345
C	0.759903	1.368451	2.469681
N	-0.013890	1.480145	1.378980
C	1.188631	-2.317237	3.466857
H	0.346908	-2.996643	3.647630
H	1.589118	-1.968560	4.423731
H	1.965384	-2.907743	2.956903
C	1.137473	2.602011	3.243521
H	1.388975	3.424717	2.561356
H	1.986507	2.398547	3.904468
H	0.301392	2.949144	3.864380
C	-0.609357	-2.658937	1.277818
C	-1.950376	-2.848573	1.698699
C	-2.565116	-4.076047	1.433432
H	-3.592150	-4.240835	1.752986
C	-1.903961	-5.087927	0.744639
H	-2.409658	-6.031572	0.536406
C	-0.602700	-4.878696	0.312900
H	-0.090533	-5.664120	-0.242953
C	0.068905	-3.677081	0.574380
C	-2.729236	-1.751654	2.407000
H	-2.446779	-0.805165	1.918617
C	-4.245860	-1.926337	2.273390
H	-4.547668	-2.061260	1.226791
H	-4.603356	-2.794284	2.845164
H	-4.765040	-1.045952	2.666886
C	-2.358193	-1.639945	3.895404
H	-1.306398	-1.371914	4.041524
H	-2.969428	-0.866814	4.382038
H	-2.546659	-2.593597	4.409566
C	1.484937	-3.520695	0.051598
H	1.921983	-2.611892	0.493719
C	2.361748	-4.718598	0.443518
H	3.391662	-4.573405	0.099088
H	2.380208	-4.859876	1.533150
H	1.993428	-5.650278	-0.006343
C	1.466113	-3.319866	-1.471497
H	2.483073	-3.154661	-1.853000
H	1.045085	-4.201555	-1.976144
H	0.855841	-2.448264	-1.747043
C	-0.751244	2.685201	1.109986
C	-0.331094	3.560911	0.082011
C	-1.134640	4.660233	-0.240731
H	-0.825081	5.323378	-1.048768
C	-2.308445	4.930622	0.450250
H	-2.918157	5.795628	0.186609
C	-2.693342	4.086655	1.484553

H	-3.610763	4.299901	2.034606
C	-1.943646	2.955207	1.825055
C	0.965122	3.373756	-0.685562
H	1.546107	2.577211	-0.194461
C	1.809328	4.656089	-0.662122
H	2.039695	4.966146	0.365885
H	2.756411	4.501248	-1.193909
H	1.290380	5.487383	-1.158212
C	0.691665	2.926652	-2.127881
H	0.106274	1.996780	-2.148981
H	0.123451	3.695788	-2.671100
H	1.632934	2.748560	-2.663507
C	-2.480917	2.054264	2.923336
H	-1.817558	1.183433	3.010316
C	-3.884535	1.551141	2.560656
H	-4.264658	0.885161	3.346034
H	-4.590449	2.387643	2.468386
H	-3.881218	1.007360	1.608062
C	-2.526493	2.762109	4.286615
H	-2.859865	2.063625	5.066957
H	-1.547190	3.158442	4.583203
H	-3.232300	3.604655	4.266124
C	4.517220	-0.302500	-1.552592
C	4.677585	-0.930274	-0.272357
C	4.646106	0.088072	0.726460
C	4.493649	1.347967	0.076591
C	4.412802	1.114456	-1.332965
C	4.623535	-0.970346	-2.889959
H	4.304336	-2.020506	-2.848374
H	4.003186	-0.469317	-3.645723
H	5.660772	-0.962642	-3.265538
C	5.004503	-2.368497	-0.030550
H	4.571154	-2.737249	0.908785
H	4.644024	-3.011642	-0.843133
H	6.094962	-2.520171	0.038433
C	4.813169	-0.120055	2.199424
H	4.388420	-1.079494	2.526254
H	5.876328	-0.118684	2.494247
H	4.315306	0.671882	2.774375
C	4.486017	2.671545	0.777971
H	4.279246	3.495955	0.087057
H	3.730115	2.713663	1.576832
H	5.459547	2.878745	1.250756
C	4.410760	2.143095	-2.421785
H	4.018929	3.107342	-2.077098
H	5.431012	2.325181	-2.799218
H	3.804113	1.829325	-3.282744
C	-2.290628	-1.193475	-3.375743
C	-2.094024	0.193280	-3.672664
C	-3.020260	0.951629	-2.886596
C	-3.798794	0.029792	-2.106997
C	-3.347608	-1.299501	-2.413614
C	-1.563737	-2.342931	-4.003424
H	-0.535158	-2.072255	-4.278162

H	-2.065598	-2.685740	-4.923562
H	-1.508702	-3.203707	-3.323385
C	-1.117573	0.739623	-4.668716
H	-0.858633	1.783884	-4.452894
H	-1.526506	0.710032	-5.691971
H	-0.182006	0.163120	-4.678358
C	-3.199267	2.439279	-2.902328
H	-4.039269	2.739347	-3.550219
H	-2.301323	2.948139	-3.274952
H	-3.401831	2.836560	-1.898021
C	-4.948208	0.398664	-1.220112
H	-5.096046	-0.333655	-0.415482
H	-5.892683	0.453482	-1.786649
H	-4.796026	1.379819	-0.750438
C	-3.927953	-2.578977	-1.894402
H	-3.152198	-3.329959	-1.691761
H	-4.635113	-3.021334	-2.615356
H	-4.476709	-2.420834	-0.957617

RC_{Si}^{H2}

74

C	-3.307674	-1.248339	-0.313930
C	-2.678022	-0.015427	-0.042384
C	-3.278584	1.212682	-0.386659
C	-4.519156	1.182356	-1.034775
C	-5.147338	-0.025048	-1.324826
C	-4.547050	-1.228421	-0.962417
N	-1.365560	-0.015236	0.561406
C	-1.255830	-0.007938	1.962475
C	-2.532193	0.010719	2.750546
C	-2.601470	2.541690	-0.098467
C	-3.534010	3.530259	0.613054
C	-2.646910	-2.568223	0.046270
C	-3.619778	-3.567894	0.682114
SI	-0.023709	-0.014716	-0.588842
N	1.299637	-0.021105	0.561357
C	2.603582	-0.031525	-0.062784
C	3.230462	1.195165	-0.361289
C	4.463152	1.162173	-1.022227
C	5.063185	-0.047769	-1.360568
C	4.440934	-1.249133	-1.034415
C	3.206001	-1.266254	-0.376140
C	2.581914	2.523141	-0.014129
C	1.996542	3.180021	-1.273625
C	2.538008	-2.588809	-0.046291
C	2.040857	-3.275827	-1.327073
C	1.264953	-0.026735	1.979049
C	-0.050870	-0.014029	2.588510
C	2.396224	-0.039564	2.732183
C	3.459036	-3.511473	0.762136
C	3.546787	3.469553	0.709866

C	-1.963904	-3.176065	-1.189135
C	-2.036154	3.148783	-1.391782
H	2.313312	-0.041636	3.816689
H	3.387782	-0.047754	2.289005
H	-0.059511	-0.007473	3.677376
H	-2.313818	0.023413	3.823284
H	-3.153407	-0.868651	2.526534
H	-3.139953	0.892877	2.502216
H	4.959703	2.098477	-1.279437
H	6.022559	-0.053968	-1.879661
H	4.919745	-2.192365	-1.300666
H	1.663052	-2.370641	0.580700
H	4.346325	-3.806551	0.184156
H	2.923794	-4.429681	1.042109
H	3.797596	-3.016317	1.681854
H	1.350552	-2.625161	-1.881929
H	1.516060	-4.211210	-1.086405
H	2.881589	-3.516150	-1.993569
H	1.751776	2.314256	0.674013
H	3.960124	2.994513	1.609411
H	3.019482	4.384306	1.014681
H	4.384347	3.770002	0.064729
H	2.790673	3.405373	-2.000076
H	1.484788	4.119278	-1.019910
H	1.273054	2.515278	-1.766920
H	-4.999448	2.119322	-1.319048
H	-6.111798	-0.029204	-1.834132
H	-5.049701	-2.168536	-1.191031
H	-1.861899	-2.355723	0.785730
H	-2.705631	-3.401437	-1.969059
H	-1.442864	-4.107432	-0.926525
H	-1.228843	-2.480320	-1.619578
H	-4.131166	-3.133162	1.551858
H	-3.073755	-4.460705	1.016355
H	-4.387661	-3.899917	-0.030364
H	-1.754840	2.345106	0.573757
H	-2.843818	3.359747	-2.107460
H	-1.330343	2.459613	-1.876664
H	-1.508832	4.089498	-1.180060
H	-4.378228	3.823660	-0.026289
H	-2.984125	4.444374	0.876052
H	-3.944199	3.099913	1.536753
H	2.157807	-0.046205	-3.109705
H	2.560212	-0.047751	-3.745551

TS_{Si}^{H2}

74

C	2.968650	-1.219044	0.692423
C	2.653318	0.062992	0.195842
C	3.559789	1.138671	0.312765
C	4.803025	0.898314	0.907670
C	5.139658	-0.367358	1.380038

C	4.226102	-1.411612	1.276584
N	1.377898	0.287776	-0.434082
C	1.228518	0.175527	-1.822864
C	2.480279	-0.043674	-2.621634
C	3.193874	2.540726	-0.142118
C	4.273205	3.182111	-1.023132
C	1.990831	-2.378196	0.622759
C	2.488691	-3.481359	-0.322431
SI	-0.020846	0.762772	0.496350
N	-1.387989	0.248676	-0.436649
C	-2.637663	-0.086825	0.190095
C	-3.488857	0.940281	0.645075
C	-4.684133	0.582170	1.281359
C	-5.039392	-0.753687	1.438444
C	-4.200022	-1.756529	0.958062
C	-2.989406	-1.447765	0.329678
C	-3.142057	2.405514	0.453898
C	-2.830862	3.085615	1.795991
C	-2.103190	-2.561778	-0.195565
C	-1.781065	-3.595812	0.890391
C	-1.315847	0.288554	-1.852850
C	0.014829	0.265223	-2.441502
C	-2.432964	0.272441	-2.624161
C	-2.733453	-3.234013	-1.425096
C	-4.248703	3.156997	-0.298822
C	1.701300	-2.938062	2.022761
C	2.889796	3.429809	1.074758
H	-2.337580	0.237121	-3.706765
H	-3.429792	0.261150	-2.191269
H	0.026581	0.236658	-3.529875
H	2.237429	-0.130622	-3.685342
H	2.994953	-0.961541	-2.302751
H	3.196423	0.778758	-2.485355
H	-5.349410	1.363181	1.652285
H	-5.975437	-1.015967	1.933325
H	-4.490022	-2.801411	1.077380
H	-1.158298	-2.102941	-0.517666
H	-3.684948	-3.718641	-1.161137
H	-2.060025	-4.004648	-1.826803
H	-2.925945	-2.495964	-2.214228
H	-1.341722	-3.119752	1.776597
H	-1.067682	-4.339140	0.507332
H	-2.682810	-4.137211	1.209558
H	-2.237016	2.452517	-0.169956
H	-4.458682	2.680391	-1.265299
H	-3.941129	4.195089	-0.486852
H	-5.182790	3.184184	0.280255
H	-3.716870	3.090121	2.447593
H	-2.521013	4.128074	1.637563
H	-2.027796	2.562496	2.333884
H	5.517041	1.716524	1.008187
H	6.114418	-0.538303	1.838592
H	4.492275	-2.397595	1.660047
H	1.046948	-1.994392	0.210801

H	2.614699	-3.327410	2.493470
H	0.977557	-3.761112	1.963893
H	1.287710	-2.161461	2.680610
H	2.657181	-3.094220	-1.336311
H	1.749475	-4.292259	-0.385940
H	3.434323	-3.912408	0.036593
H	2.271605	2.470568	-0.736902
H	3.786563	3.555498	1.698648
H	2.107569	2.988617	1.708102
H	2.552979	4.424570	0.751479
H	5.204647	3.349733	-0.464504
H	3.927730	4.157808	-1.391327
H	4.511183	2.553880	-1.892150
H	0.031268	2.131042	1.121175
H	0.003931	2.416900	-0.105975

PC_{Si}^{H2}

74

C	-3.248411	-1.230357	-0.477444
C	-2.652878	0.005402	-0.137733
C	-3.272705	1.232481	-0.464403
C	-4.503744	1.197324	-1.129288
C	-5.105305	-0.012048	-1.465135
C	-4.480556	-1.212914	-1.140434
N	-1.362548	0.014077	0.499043
C	-1.224293	-0.017501	1.891843
C	-2.496441	-0.039936	2.685586
C	-2.643559	2.571112	-0.117762
C	-3.537104	3.390309	0.825671
C	-2.587635	-2.559105	-0.152443
C	-3.473006	-3.429008	0.752176
SI	0.020600	0.057115	-0.562720
N	1.379074	0.014831	0.511114
C	2.659170	0.004865	-0.142235
C	3.277211	1.230860	-0.471901
C	4.499733	1.194111	-1.152771
C	5.095982	-0.017154	-1.492861
C	4.477811	-1.217792	-1.152745
C	3.254764	-1.232328	-0.472981
C	2.661817	2.565395	-0.091655
C	2.384597	3.436616	-1.324838
C	2.610329	-2.554791	-0.099046
C	2.295789	-3.403679	-1.338993
C	1.309708	-0.014122	1.918330
C	-0.014855	-0.027456	2.517507
C	2.429653	-0.032599	2.689317
C	3.483127	-3.332369	0.897101
C	3.542264	3.307961	0.924388
C	-2.211841	-3.321650	-1.432496
C	-2.313418	3.378312	-1.382227
H	2.332274	-0.060187	3.771675
H	3.427313	-0.022092	2.258481

H	-0.027314	-0.048180	3.605483
H	-2.273309	-0.045676	3.756968
H	-3.100543	-0.926590	2.445980
H	-3.123376	0.833973	2.458632
H	4.993213	2.130145	-1.418762
H	6.048690	-0.025666	-2.024150
H	4.953941	-2.162622	-1.419446
H	1.660989	-2.326402	0.403590
H	4.451770	-3.599966	0.450336
H	2.981317	-4.263008	1.197592
H	3.670937	-2.734615	1.798425
H	1.652360	-2.857545	-2.041600
H	1.777473	-4.328002	-1.046810
H	3.214851	-3.688218	-1.871212
H	1.700921	2.356664	0.397383
H	3.701609	2.695647	1.821455
H	3.062577	4.249064	1.228378
H	4.524001	3.552477	0.493433
H	3.317230	3.706753	-1.840579
H	1.883492	4.368847	-1.028086
H	1.738654	2.914466	-2.043217
H	-4.998487	2.134319	-1.389288
H	-6.064518	-0.018864	-1.984313
H	-4.956735	-2.156771	-1.409974
H	-1.659699	-2.343714	0.394773
H	-3.109109	-3.591433	-2.007780
H	-1.679549	-4.249862	-1.181290
H	-1.562051	-2.717886	-2.078971
H	-3.737273	-2.908167	1.682205
H	-2.947897	-4.356758	1.018457
H	-4.408220	-3.705907	0.245112
H	-1.700513	2.368047	0.407842
H	-3.227851	3.638860	-1.934231
H	-1.658992	2.811305	-2.056793
H	-1.802744	4.314050	-1.114682
H	-4.491544	3.646490	0.344168
H	-3.037136	4.328746	1.102741
H	-3.763843	2.839845	1.748443
H	0.018791	-1.111046	-1.491331
H	0.009973	1.292648	-1.400558

RC_{Al}^{H2}

75

C	-4.377262	-1.650274	-0.837916
C	-5.275986	-0.606490	-1.045339
C	-4.895208	0.703522	-0.770530
C	-3.626795	0.994549	-0.255908
C	-2.746309	-0.078845	-0.006176
C	-3.096659	-1.411157	-0.327801
C	-3.194776	2.437229	-0.051938
C	-4.271337	3.302799	0.612527

N	-1.427277	0.171751	0.510738
C	-1.282612	0.369311	1.830411
C	-0.026344	0.522807	2.435347
C	1.226409	0.409560	1.819105
C	2.449544	0.464104	2.695214
C	-2.106709	-2.549458	-0.129517
C	-2.078174	-3.044455	1.324628
C	-2.495203	0.423896	2.723359
AL	-0.035167	0.178284	-0.923387
N	1.368561	0.236392	0.494957
C	2.691341	0.011946	-0.027639
C	3.141356	-1.316984	-0.186464
C	4.391190	-1.527820	-0.779169
C	5.175353	-0.458838	-1.203475
C	4.718078	0.845879	-1.035005
C	3.475411	1.107522	-0.447878
C	2.299337	-2.505301	0.250297
C	1.784052	-3.288014	-0.966887
C	2.983352	2.538114	-0.305921
C	4.027223	3.457311	0.341526
C	2.529598	3.088825	-1.667069
C	-2.322131	-3.717527	-1.093493
C	-2.750760	3.041568	-1.394644
C	3.055741	-3.425042	1.218284
H	2.102345	2.522557	0.350785
H	-3.064012	1.346622	2.540640
H	-2.203228	0.400645	3.778113
H	-3.178572	-0.409394	2.515787
H	-1.104648	-2.137615	-0.351439
H	-1.942017	2.447157	-1.844820
H	-2.389320	4.070517	-1.255673
H	-3.589444	3.062315	-2.105405
H	-0.024029	0.681068	3.511059
H	3.021441	-0.471157	2.616844
H	2.176293	0.627582	3.742391
H	3.124945	1.267282	2.370247
H	-2.315349	2.436671	0.606405
H	3.370070	3.114519	-2.375673
H	2.137462	4.110304	-1.560152
H	1.740108	2.458344	-2.101731
H	-6.270198	-0.814353	-1.442786
H	5.337137	1.678493	-1.371389
H	-3.246710	-4.268339	-0.869463
H	-1.489313	-4.428170	-1.005434
H	-2.369963	-3.373492	-2.135147
H	4.754135	-2.547635	-0.915017
H	4.366211	3.063381	1.309762
H	3.599516	4.455518	0.508771
H	4.911768	3.579545	-0.298993
H	1.422527	-2.114842	0.785594
H	-5.593747	1.517250	-0.967602
H	-5.147474	3.432476	-0.037829
H	-3.870617	4.303221	0.825988
H	-4.617893	2.863126	1.558156

H	3.925778	-3.890469	0.734017
H	2.397147	-4.232397	1.567758
H	3.415810	-2.873162	2.097459
H	6.145007	-0.642557	-1.667852
H	-4.674457	-2.667963	-1.088470
H	-1.793443	-2.247016	2.021094
H	-1.351588	-3.862291	1.433630
H	-3.066776	-3.423958	1.621584
H	1.198065	-2.636742	-1.632142
H	1.144174	-4.122653	-0.646298
H	2.619182	-3.701806	-1.550158
H	-0.183841	1.909084	-3.836383
H	-0.284343	2.358119	-4.435248

TS_{Al}^{H2}

75

C	-3.578453	-1.000556	-0.556339
C	-2.724028	0.030618	-0.116423
C	-3.102090	1.388090	-0.201679
C	-4.365240	1.691344	-0.720827
C	-5.227657	0.685540	-1.148934
C	-4.831954	-0.646455	-1.069201
N	-1.415226	-0.296036	0.388560
AL	0.011863	-0.495440	-0.853161
N	1.441259	-0.216927	0.373142
C	1.287302	-0.376221	1.694655
C	2.488809	-0.298304	2.595863
C	-2.171342	2.510512	0.225331
C	-2.778546	3.370714	1.342509
C	-3.156148	-2.458503	-0.520633
C	-2.917896	-2.983494	-1.945127
C	2.733281	0.103560	-0.172525
C	3.688987	-0.916189	-0.358475
C	4.941993	-0.558546	-0.871244
C	5.231570	0.758636	-1.209748
C	4.255151	1.743501	-1.070995
C	2.989574	1.439307	-0.560616
C	3.363966	-2.377641	-0.101866
C	3.145755	-3.103352	-1.440046
C	1.911449	2.504499	-0.441137
C	2.072553	3.643381	-1.450562
C	4.434397	-3.092675	0.732004
C	0.033215	-0.593357	2.294738
C	-1.235722	-0.469826	1.707148
C	-2.439139	-0.495105	2.607933
C	1.800731	3.064672	0.985455
C	-1.773512	3.375764	-0.979352
C	-4.168188	-3.339864	0.223324
H	-2.194596	-2.521428	0.010231
H	3.076241	-1.224705	2.521823
H	2.185680	-0.163798	3.638692

H	3.154209	0.521359	2.297844
H	0.951481	2.004843	-0.673966
H	2.356000	-2.617694	-2.027925
H	2.848494	-4.146489	-1.264174
H	4.068349	-3.101560	-2.038769
H	0.044269	-0.742945	3.371483
H	-3.011896	0.437955	2.519121
H	-2.144442	-0.635338	3.651994
H	-3.119635	-1.305792	2.312627
H	2.410641	-2.427786	0.443113
H	-3.849837	-2.969394	-2.529082
H	-2.546801	-4.017245	-1.913563
H	-2.176666	-2.368743	-2.473611
H	6.214290	1.018093	-1.605436
H	-5.506456	-1.429650	-1.417769
H	2.949043	4.266925	-1.224091
H	1.191191	4.296913	-1.417746
H	2.177164	3.261531	-2.474680
H	-4.676479	2.734491	-0.795142
H	-4.351099	-2.974689	1.243379
H	-3.793159	-4.370307	0.292287
H	-5.134812	-3.371399	-0.299171
H	-1.254633	2.053440	0.622325
H	5.698295	-1.330246	-1.019288
H	5.386836	-3.165577	0.188362
H	4.108565	-4.115616	0.964834
H	4.631049	-2.572237	1.679406
H	-3.687651	3.884953	0.999919
H	-2.060728	4.137400	1.666337
H	-3.045918	2.763839	2.218125
H	-6.207993	0.941333	-1.552723
H	4.481156	2.764982	-1.374061
H	1.583942	2.280168	1.719933
H	0.993540	3.809089	1.039857
H	2.739118	3.558236	1.276960
H	-1.296597	2.768528	-1.761285
H	-1.068652	4.160733	-0.671498
H	-2.652435	3.866478	-1.420724
H	0.084836	-2.424458	-0.684972
H	0.062024	-1.773413	-1.808251

PC_{Al}^{H2}

75

C	-3.546433	-1.102257	-0.576520
C	-2.720250	-0.054967	-0.116289
C	-3.113791	1.296838	-0.237736
C	-4.356271	1.576388	-0.815890
C	-5.185931	0.554949	-1.270839
C	-4.778998	-0.770345	-1.151714
N	-1.421582	-0.352520	0.424128
AL	0.013763	-0.607262	-0.825150

N	1.425207	-0.244350	0.431472
C	1.264268	-0.363599	1.755035
C	2.464195	-0.297487	2.661101
C	-2.222239	2.440857	0.216455
C	-2.906318	3.320918	1.272243
C	-3.135369	-2.561183	-0.471298
C	-2.934978	-3.178407	-1.863122
C	2.720308	0.045917	-0.120939
C	3.654246	-0.992248	-0.325748
C	4.895836	-0.662667	-0.883480
C	5.201546	0.646140	-1.239313
C	4.254600	1.652701	-1.062496
C	2.998717	1.376586	-0.513106
C	3.339414	-2.444654	-0.007473
C	3.158747	-3.251434	-1.303771
C	1.969109	2.479961	-0.327459
C	2.045303	3.558066	-1.412048
C	4.407984	-3.094136	0.883386
C	0.005404	-0.544761	2.358156
C	-1.257399	-0.455779	1.750193
C	-2.470154	-0.441584	2.639972
C	2.065141	3.111336	1.070357
C	-1.764512	3.284951	-0.981985
C	-4.144978	-3.380223	0.346048
H	-2.168052	-2.600726	0.048502
H	2.996838	-1.259379	2.644602
H	2.163614	-0.090569	3.692820
H	3.178007	0.464325	2.325877
H	0.978618	2.008730	-0.409502
H	2.347503	-2.836023	-1.914528
H	2.909391	-4.296381	-1.070812
H	4.083322	-3.244434	-1.899011
H	0.007831	-0.652669	3.439959
H	-3.007165	0.512177	2.546413
H	-2.190383	-0.588543	3.687322
H	-3.177755	-1.226257	2.339348
H	2.381959	-2.475235	0.530457
H	-3.871817	-3.165147	-2.438729
H	-2.604876	-4.222902	-1.772967
H	-2.172483	-2.630126	-2.430942
H	6.174939	0.882708	-1.670858
H	-5.429917	-1.567399	-1.513941
H	2.966393	4.153332	-1.336731
H	1.200725	4.251506	-1.307133
H	2.001622	3.116408	-2.416345
H	-4.676514	2.614418	-0.917971
H	-4.296895	-2.954253	1.347298
H	-3.788923	-4.413027	0.464782
H	-5.123821	-3.418782	-0.152625
H	-1.324735	2.006304	0.676767
H	5.632854	-1.449925	-1.047258
H	5.369170	-3.178501	0.357193
H	4.096705	-4.108669	1.168020
H	4.582033	-2.517671	1.801980

H	-3.792282	3.823645	0.858984
H	-2.214385	4.098488	1.624951
H	-3.230021	2.733791	2.142419
H	-6.149450	0.793128	-1.722975
H	4.495693	2.671174	-1.365177
H	1.906909	2.369860	1.863166
H	1.303761	3.896083	1.186891
H	3.053526	3.568956	1.222523
H	-1.224413	2.667859	-1.711595
H	-1.097601	4.091950	-0.647276
H	-2.623926	3.746614	-1.488836
H	0.090436	-2.137985	-1.300998
H	-0.037678	0.501933	-1.979787
